# Insights on Osmotic Tolerance Mechanisms in *Escherichia coli* Gained from an *rpoC* Mutation

**DOI:** 10.3390/bioengineering4030061

**Published:** 2017-06-28

**Authors:** Yuqi Guo, James Winkler, Katy C. Kao

**Affiliations:** 1 Department of Chemical Engineering, Texas A&M University, College Station, TX 77843, USA; guo.yuqi@outlook.com; 2 Department of Chemical and Biological Engineering, University of Colorado-Boulder, Boulder, CO 80303, USA; james.winkler@gmail.com

**Keywords:** *E. coli*, osmotic tolerance, *rpoC*, amino acid, acetic acid, membrane integrity, complex phenotype

## Abstract

An 84 bp in-frame duplication (K370_A396dup) within the rpoC subunit of RNA polymerase was found in two independent mutants selected during an adaptive laboratory evolution experiment under osmotic stress in *Escherichia coli*, suggesting that this mutation confers improved osmotic tolerance. To determine the role this mutation in *rpoC* plays in osmotic tolerance, we reconstructed the mutation in BW25113, and found it to confer improved tolerance to hyperosmotic stress. Metabolite analysis, exogenous supplementation assays, and cell membrane damage analysis suggest that the mechanism of improved osmotic tolerance by this *rpoC* mutation may be related to the higher production of acetic acid and amino acids such as proline, and increased membrane integrity in the presence of NaCl stress in exponential phase cells. Transcriptional analysis led to the findings that the overexpression of methionine related genes *metK* and *mmuP* improves osmotic tolerance in BW25113. Furthermore, deletion of a stress related gene *bolA* was found to confer enhanced osmotic tolerance in BW25113 and MG1655. These findings expand our current understanding of osmotic tolerance in *E. coli*, and have the potential to expand the utilization of high saline feedstocks and water sources in microbial fermentation.

## 1. Introduction

One of the challenges in the microbial production of chemicals is the low tolerance of the microbial host to inhibitors present in the feedstock. There is increasing interest in using more sustainable feedstocks such as lignocellulosic hydrolysates [1,2,3] and waste glycerol [4,5]. However, some of these feedstocks contain inhibitory components, such as high salts, that can negatively impact the performance of the microbial hosts, leading to reduced productivity [6,7]. In addition, the use of seawater or wastewater as a replacement for freshwater in fermentation can help to alleviate freshwater demand in industrial biotechnology. However, partly due to the high salinity of these water sources and low osmotic tolerance of microbial hosts [8,9], their adoption has not been widespread. Thus, improved osmotic tolerance in microbial systems can potentially increase their productivity when using high saline feedstocks, and expand the utilization of these alternate feedstocks and water sources.

As with other complex phenotypes, osmotic tolerance involves multiple genes and mechanisms. Existing studies have identified several cellular responses to osmotic stress in *Escherichia coli*. In response to increased osmotic pressure, the synthesis of aquaporin increases to accelerate the export of water to balance intracellular and environmental osmolality [10], the intracellular potassium concentration increases via the regulation of potassium transporters [11,12], and the accumulation of osmoprotectants (e.g., trehalose, glycine betaine, proline, etc.) also increases [13,14,15,16]. In addition to the regulation of intracellular osmolality, *E. coli* has been found to counter osmotic stress by accumulating ubiquinone-8 to enhance its cytoplasmic membrane stability [17]. Based on existing knowledge, there have been prior attempts to rationally improve osmotolerance in *E. coli* [18,19]. However, the levels of improvement [20,21,22,23] achieved have been modest, potentially due to limited knowledge of the genetic determinants and mechanisms involved.

We had previously identified an 84 bp in-frame duplication (K370_A396dup) in the RNA polymerization domain of RpoC of the RNA polymerase in two independently evolved osmotolerant mutants [24], which suggests that this *rpoC* mutation confers osmotic tolerance and warrants further study. We reconstructed this *rpoC* mutation in a wild-type BW25113 background and confirmed the beneficial effect of this mutation on osmotic tolerance. The reconstructed *rpoC* mutant exhibited improved growth in minimal media under osmotic stress compared with the wild-type strain. Since the *rpoC* mutation was selected during evolution in media supplemented with tryptophan [24], the impact of tryptophan supplementation was also investigated and found to confer increased osmotic tolerance, which has not been reported previously. Subsequent metabolite analysis, membrane damage analysis and transcriptional analysis of the mutant revealed potential mechanisms of how this specific *rpoC* mutation confers tolerance to osmotic stress.

## 2. Materials and Methods

### 2.1. Media and Growth Conditions

M9 minimal media (per liter: 12.8 g Na_2_HPO_4_·7H_2_O, 3 g KH_2_PO_4_, 0.5 g NaCl, 1 g NH_4_Cl, 10 mg FeCl_3_·6H_2_O, 1.8 mg ZnSO_4_·7H_2_O, 1.2 mg CuCl_2_·2H_2_O, 1.2 mg MnSO_4_·H_2_O, 1.8 mg CoCl_2_·6H_2_O) supplemented with 0.5% (*w*/*v*) glucose and Luria–Bertani (LB) broth were used for routine cultivation and growth assays. LB agar plates were used for strain isolation. Tryptophan (50 µg mL^−1^), kanamycin (10 µg mL^−1^), ampicillin (30 µg mL^−1^) and chloramphenicol (25 µg mL^−1^) were supplemented whenever necessary. All liquid cultures were cultivated with agitation at 37 °C. Sodium chloride (NaCl) was utilized to adjust the osmotic strength of the media for growth assays. The starting pH of all the media used in this work was ~7.

### 2.2. Marker-Less Reconstruction of rpoC Mutation in BW25113

In order to construct a marker-less *rpoC* K370_A396dup mutation in the wild-type BW25113 strain (F-, Δ*(araD-araB)567*, *lacZ4787(del)::rrnB-3*, *λ-*, *rph-1*, Δ*(rhaD-rhaB)568*, *hsdR514*), an *∆argE744::kan* cassette, which resides 30,503 bp away from the *rpoC* gene in the chromosome, from JW3929 of the Keio collection [25] was transduced via P1 transduction [26] into the evolved osmotolerant mutant G3 [24] containing the *rpoC* K370_A396dup mutation. The transductants with *∆argE744::kan* cassette were selected on LB agar plate with 10 µg mL^−1^ kanamycin. The kanamycin-resistant transductant containing the mutated *rpoC* allele was verified by colony PCR. The primers used for the colony PCR verification are: GAA ACC AAC TCC GAA ACC AA (forward) and AGT ACC GGT TCA AAT GCC TG (reverse). The transductant containing both the Δ*argE744::kan* cassette and the mutated *rpoC* allele was named EJW1. The Δ*argE744::kan-rpoC* K370_A396dup construct from EJW1 was then transduced into BW25113 selected for kanamycin resistance, and the same colony PCR screening procedure was used to isolate transductant EJW2, which contains both Δ*argE744::kan* and *rpoC* K370_A396dup. Then EJW2 was made arginine prototrophic (arg+) via P1 transduction with the wild-type *argE* allele from BW25113 and selected on M9 minimal agar plate for arginine prototroph. The arg+ colonies that contain the *rpoC* K370_A396dup mutation was verified using the same colony PCR screening procedure. The resulting reconstructed mutant EJW3 therefore contains only the *rpoC* mutation from mutant G3 without any added markers. Since the original G3 mutant contained ∆*trpB769::kan* deletion, EJW3 was transduced with ∆*trpB769::kan* cassette from JW1253 of the Keio collection and selected on an LB agar plate with 10 µg mL^−1^ kanamycin to create a tryptophan auxotroph, EJW4, similar to the one in the original G3 mutant. The same method was used to introduce this *rpoC* mutation in strain MG1655 (F-, *λ-*, *rph-1*), and the resulting MG1655 *rpoC* mutant was named EYG1. All strains used in this study are listed in Table 1.

### 2.3. Growth Kinetic Analysis

Frozen stocks of strains were streaked on LB agar plates for single colonies and incubated overnight at 37 °C. Single colonies were used to inoculate 5 mL of fresh M9 media and incubated overnight at 37 °C with shaking (225 rpm). The overnight cultures were washed once with fresh M9 media and re-suspended in M9 to an OD_600_ of ~10.0, then 50 µL samples were inoculated in 5 mL M9 media supplemented with 0.6 M NaCl in 20 × 150 mm screw-capped test tubes, at an initial OD_600_ of approximately 0.1. The media was supplemented with 50 µg mL^−1^ tryptophan when necessary. Samples were incubated at 37 °C with shaking (225 rpm), and growth was tracked by measuring the OD_600_ periodically using a spectrophotometer (BioMate 3, Thermo Fisher Scientific, Madison, WI, USA). 

For viability assays, samples were plated from cultures sampled at different growth phases on LB plates. After overnight incubation at 37 °C, colony forming units (CFU) were counted to estimate the concentration of viable cells. The size and shape of cells at different growth phases in M9 and M9 supplemented with 0.6 M NaCl were observed under a light microscope (Carl Zeiss, Göttingen, Germany). 

The ability of each strain to grow under higher osmotic stress was analyzed, as described previously, with an initial OD_600_ of ~0.05 and agitated at 275 rpm (a higher agitation was used to prevent cell clumping in the presence of higher osmotic stress). Screw-capped test tubes (20 × 150 mm) were used to analyze cell growth under micro-aerobic condition in M9 with addition of 0.8 M, 0.9 M, or 1 M NaCl. Normal test tubes (20 × 150 mm) were used to analyze cell growth under aerobic condition in M9 with addition of 0.75 M, 0.8 M, or 0.9 M NaCl. Three biological replicates were used in each condition. 

### 2.4. Effects of Amino Acid Supplementation

The effects of individual amino acid supplementation on osmotic tolerance were measured in M9 in the presence of 0.65 M NaCl. The same procedure for growth kinetic analysis as described above was carried out using 5 mL cultures. Three biological replicates were used in each condition.

### 2.5. Metabolite Analysis

Cells for metabolite analysis were cultivated in two 50 mL cultures with M9 media or M9 media supplemented with 0.6 M NaCl in 250 mL screw-capped flasks, incubated at 37 °C with shaking (275 rpm). Samples for metabolite analysis were collected during late exponential phase (OD_600_ about 0.7–1.0), and analyzed using high-performance liquid chromatography (HPLC; Agilent Technologies, 1260 Infinity, Santa Clara, CA, USA). Extracellular metabolites were collected from supernatants, and intracellular metabolites were extracted with acetonitrile:methanol:water (40:40:20) [27] from cell pellets collected from combining two 50 mL cultures inoculated with the same overnight culture originated from a single colony. Trehalose, glucose, and acetic acid were analyzed using Aminex HPX-87H ion exclusion column (Bio-Rad, Hercules, CA, USA) operated at 50 °C with 5 mM sulfuric acid as the mobile phase at a flow rate of 0.6 mL min^−1^, and detected by Refractive Index (RI) detector. Free amino acids were analyzed using a modified Agilent amino acid analysis method [28]; the detailed procedure of which can be found in the Supplementary Materials (Tables S1, S2 and S3). Six biological replicates were used in each condition.

### 2.6. Effects of Acetic Acid Supplementation

For experiments with acetic acid supplementation, 10 mM of acetic acid was used. The starting pH of the media with 10 mM acetic acid addition was ~6.5. Cells were cultivated as described in the metabolite analysis section above with 5 mL media in screw-capped tubes. Three biological replicates were used in each condition.

### 2.7. Cell Membrane Damage Analysis

Cell membrane perturbation was analyzed by measuring the uptake of the fluorescent dye propidium iodide (PI). The OD_600_ of cells grown in M9 was adjusted to ~0.6, treated with M9 only, M9 supplemented with 0.7 M NaCl, M9 supplemented with 10 mM acetic acid, or M9 supplemented with 0.7 M NaCl and 10 mM acetic acid for 30 min at 37 °C. The treated cells were centrifuged at 21,130× *g* for 2 min, and pellets were re-suspended in PBS. PI staining was performed as previously described [29,30]. Briefly, PI was added to cells at a final concentration of 2.9 µM and incubated in the dark for 10 min at room temperature, then the cells were washed twice with PBS. To quantify uptake of PI, 200 µL of each sample was placed in black-walled 96-well plates (Greiner Bio-One, Monroe, NC, USA) and the fluorescence of PI staining was measured using a fluorescent plate reader (Molecular Devices SpectraMax® Gemini EM, Sunnyvale, CA, USA) using an excitation wavelength of 495 nm and an emission wavelength of 615 nm. The background fluorescence was corrected by subtracting the fluorescence of cells without PI straining. All fluorescence data were normalized by OD_600_. Three biological replicates were used in each condition.

### 2.8. Transcriptional Analysis

Perturbations to transcriptional regulation in the presence of 0.6 M NaCl were determined using microarray analysis using strains BW25113, EJW3, MG1655, and EYG1 with two biological replicates each. Samples were cultivated using the same condition as in metabolite analysis with 25 mL cultures and an initial OD_600_ of ~0.05. When OD_600_ reached ~0.5, cells were quickly chilled to ≤4 °C on dry ice/isopropanol bath, then harvested by centrifugation (4470× *g*) at 4 °C followed by immediate re-suspension in 5 mL of RNAlater (Sigma-Aldrich, St. Louis, MO, USA). Total RNA was extracted by using the ZR Fungal/Bacterial RNA MicroPrep™ (Zymo Research, Irvine, CA, USA) kit. A total of 10 μg of the isolated total RNA was mixed with 1.5 μg random primers (Promega, Madison, WI, USA), incubated at 70 °C for 10 min and then cooled on ice (4 °C). The cDNA was synthesized by combining the total RNA mixture with 10 U SuperScript® III reverse transcriptase, 1× first strand buffer, 0.01 M DTT (Invitrogen, Carlsbad, CA, USA), and nucleotides (0.5 mM dATP, 0.5 mM dGTP, 0.5 mM dCTP, 0.2 mM dTTP (Promega, Madison, WI, USA) and 0.3 mM amino-allyl dUTP (Thermo Fisher Scientific, Waltham, MA, USA)), and incubated at 42 °C for 3 h. The cDNA was recovered with ice-cold ethanol precipitation, labeled with either Cy3- or Cy5- mono-reactive dye (GE Healthcare, Little Chalfont, Buckinghamshire, UK), and then hybridized to the *E. coli* Gene Expression Microarray (Agilent Technologies). The arrays were scanned using the GenePix 4100A Microarray Scanner and the images were analyzed using GenePix Pro 6.0 software (Molecular Devices, Sunnyvale, CA, USA). The MIDAS software package (TM4) [31] was used to normalize the data using LOWESS based normalization algorithm [32]. The rank product method with a critical *p*-value < 0.01 was used to identify the differentially expressed genes using MeV (TM4) microarray analysis software [31]. Gene ontology analysis was performed using the Database for Annotation, Visualization and Integrated Discovery (DAVID) [33,34].

### 2.9. Overexpression and Deletion Assay

Genes uniquely upregulated or downregulated in the *rpoC* mutant in the BW25113 background were further investigated via overexpression or deletion studies. Plasmids from the ASKA collection [35] were transformed into BW25113 for overexpression assays. The growth kinetics of the overexpression strains were compared against the wild-type strain expressing the empty vector pCA24N in M9 with or without 0.55 M NaCl supplementation. Knockout strains were obtained from the Keio collection [25]. The kanamycin resistance marker in the Keio strains was removed by transforming with the plasmid pCP20 as previously described [36], and the marker-less knockout strains were used for deletion assay using BW25113 as the negative control in M9 with or without 0.6 M NaCl supplementation. Three biological replicates were used in each condition. 

### 2.10. Microarray Data Accession Number

Microarray data have been deposited in the Gene Expression Omnibus (GEO) database with accession number GSE94342.

## 3. Results and Discussion

### 3.1. The rpoC K370_A396dup Mutation Confers Osmotic Tolerance in BW25113

Our prior work investigating osmotic tolerance in *E. coli* using adaptive laboratory evolution yielded several mutants with significantly increased tolerance to NaCl stress [24]. Two isolated osmotolerant mutants (G3 and G4) share an identical *rpoC* K370_A396dup mutation. These mutants were isolated from independent populations, which led us to hypothesize that this specific mutation is a causative mutation for the improved osmotic tolerance observed. Since both G3 and G4 also contain additional mutations, in order to study the specific function of the *rpoC* K370_A396dup mutation, we reconstructed this mutation in the BW25113 wild-type background to generate strain EJW3. In addition, since the parental strain from which G3 and G4 mutants were derived contains a *trpB* deletion, we also generated strain EJW4 by deleting the *trpB* gene from EJW3.

**Figure 1:**
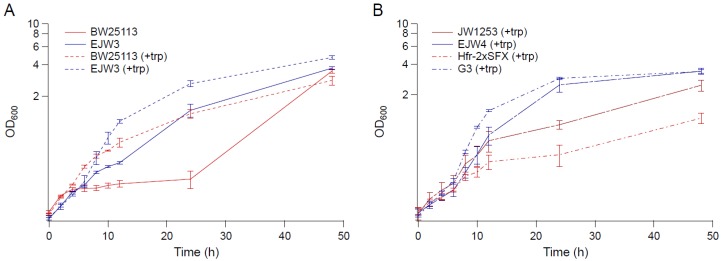
Growth kinetics in M9 supplemented with 0.6 M NaCl. (**A**) BW25113 and EJW3 in M9 supplemented with 0.6 M NaCl (solid lines: without tryptophan, and dashed lines: with 50 µg mL^−1^ tryptophan supplementation). (**B**) Tryptophan auxotrophic strains JW1253, EJW4, HFr-2×SFX- and G3 in M9 supplemented with 0.6 M NaCl and 50 µg mL^−1^ tryptophan. Red lines: strains without *rpoC* mutation. Blue lines: strains with *rpoC* mutation. Error bars are standard deviations.

The growth kinetics of strain BW25113, EJW3 (BW25113 *rpoC* K370_A396dup), JW1253 (BW25113 Δ*trpB769::kan*), EJW4 (BW25113 *rpoC* K370_A396dup, Δ*trpB769::kan*), G3 (evolved osmotolerant mutant containing *rpoC* K370_A396dup mutation, BW25113 background), and Hfr-2×SFX- (ancestor of G3, BW25113 background) [24] were compared in M9 supplemented with 0.6 M NaCl and 50 µg mL^−1^ tryptophan (Figure 1). Since BW25113 and EJW3 are prototrophic for tryptophan, we also compared the growth of these two strains in the absence of tryptophan to assess the impact of the *rpoC* K370_A396dup mutation on osmotic tolerance in the absence of amino acid supplementation. The results showed that in the absence of tryptophan supplementation, the growth of BW25113 was drastically inhibited by 0.6 M NaCl 4 h after incubation with no significant growth observed within the next 20 h, while strain EJW3 continued to grow and reached significantly higher biomass than BW25113 after 24 h. The data showed that the addition of tryptophan significantly increased the growth of both BW25113 and the reconstructed *rpoC* mutant EJW3 in the presence of 0.6 M NaCl (Figure 1A). Though the addition of tryptophan improved the performance of all strains tested in the presence of 0.6 M NaCl, strains with the *rpoC* K370_A396dup mutation (EJW3, EJW4, and G3) still outperformed their *rpoC* wild-type counterparts (Figure 1A,B), strongly suggesting that the *rpoC* K370_A396dup mutation is a causative mutation for enhanced osmotic tolerance. Interestingly, the level of osmotic tolerance conferred by the *rpoC* mutation alone was on the same level as that conferred by the addition of 50 µg mL^−1^ tryptophan. Results in Figure 1 showed no significant difference in specific growth rates during the first few hours of growth between BW25113 and EJW3; therefore, biomass concentrations after 24 and 48 h were used to assess osmotic tolerance for the majority of this work.

**Figure 2:**
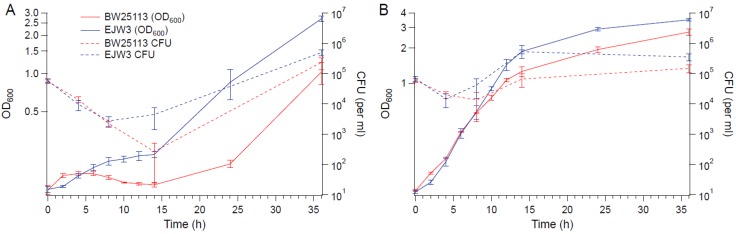
Growth and viability curves. (**A**) M9 with 0.6 M NaCl. (**B**) M9 with 0.6 M NaCl and tryptophan (50 µg mL^−1^). Error bars are standard deviations.

To determine the impact of the *rpoC* mutation on cell viability in hyperosmotic stress, BW25113 and EJW3 were grown in M9 supplemented with 0.6 M NaCl and the concentration of viable cells were quantified over time (Figure 2). The concentration of viable cells of both strains decreased within the first few hours, indicating the occurrence of cell death upon initial exposure to hyperosmotic stress. However, increases in the concentration of viable cells were observed in EJW3 sooner and at a faster rate than BW25113. The same trend was also observed using OD_600_, indicating that OD_600_ and the concentration of viable cells are correlated. The increase in growth (based on OD_600_ measurements) observed in hyperosmotic conditions when tryptophan was supplemented also correlated with increased cell viability. Furthermore, there was no observable differences in cell size or shape between BW25113 and EJW3 in the presence or absence of 0.6 M NaCl at different growth phases (Figure S1). Thus, OD_600_ was used as the measure of cell growth for the remainder of this study.

The impact of the *rpoC* mutation on tolerance to higher osmotic stress was tested in both aerobic and micro-aerobic conditions. Preliminary tests showed higher osmotic tolerance of the strains in micro-aerobic vs. aerobic conditions, therefore 0.8 M, 0.9 M, and 1 M NaCl were used in micro-aerobic conditions, while 0.75 M, 0.8 M, and 0.9 M NaCl were used under aerobic conditions. The higher osmotic tolerance observed under micro-aerobic conditions is likely due to higher induction of the osmotic tolerance related genes *ompC* and *proU* under anaerobic conditions [37]. Relative growth results in 0.8 M (micro-aerobic) and 0.75 M (aerobic) NaCl are shown in Table 2 and Table 3. In micro-aerobic and aerobic conditions, all strains with the *rpoC* mutation showed significant growth in the presence of 0.8 M and 0.75 M NaCl respectively, while only slight growth was observed in their wild-type counterparts. In the presence of 0.9 M NaCl in micro-aerobic condition and 0.8 M NaCl in aerobic condition, only slight turbidity was observed in all strains (Tables S4 and S5), and no growth was observed in any strain cultured micro-aerobically in the presence of 1 M NaCl or aerobically with 0.9 M NaCl. 

### 3.2. Effects of Amino Acid Supplementation

**Figure 3:**
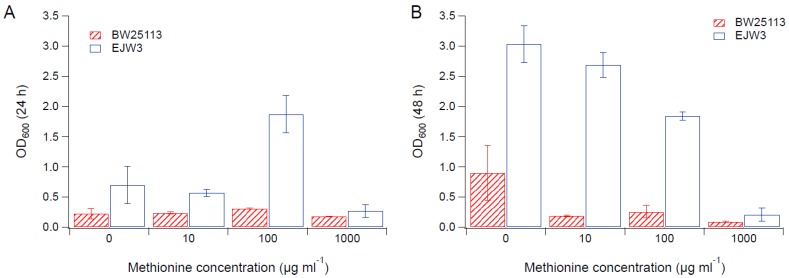
Cell density in M9 supplemented with 0.65 M NaCl and methionine. (**A**) Cell density after 24 h. (**B**) Cell density after 48 h. Error bars are standard deviations.

**Figure 4:**
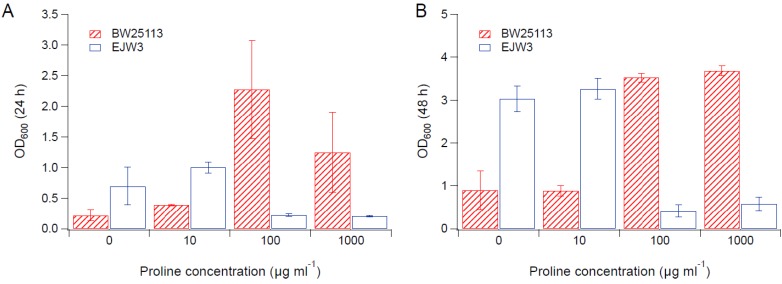
Cell density in M9 supplemented with 0.65 M NaCl and proline. (**A**) Cell density after 24 h. (**B**) Cell density after 48 h. Error bars are standard deviations.

**Figure 5:**
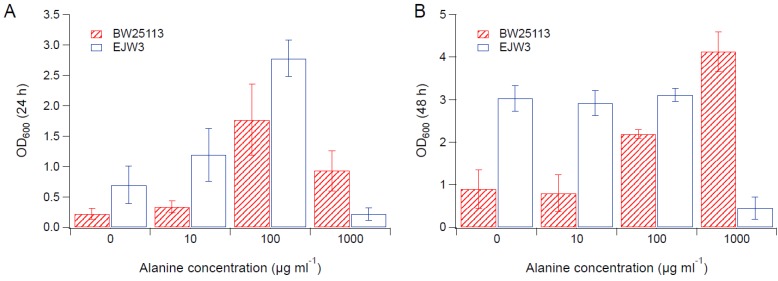
Cell density in M9 supplemented with 0.65 M NaCl and alanine. (**A**) Cell density after 24 h. (**B**) Cell density after 48 h. Error bars are standard deviations.

Since we observed an improvement in growth performance under osmotic stress with tryptophan supplementation, and prior work have reported the impact of some amino acids (e.g., glutamic acid, proline) on osmotic stress response [15,38], we hypothesized that there are additional amino acids that also play a role in osmotic tolerance in *E. coli*. Thus, we assessed the impact of individual amino acid supplementation on osmotic tolerance of BW25113 and EJW3. Preliminary data with tryptophan supplementation showed a more observable benefit in the presence of higher NaCl concentration, therefore a higher osmotic stress with 0.65 M NaCl was used. The summary of amino acid supplementation on optical cell density (OD_600_) is listed in Table S6. With a few exceptions (valine, methionine, proline, alanine), the addition of 10 µg mL^−1^ or 100 µg mL^−1^ of most amino acids tested significantly improved the growth of both BW25113 and EJW3 under osmotic stress. This may be partially due to the decreased ATP requirements for amino acid biosynthesis when these amino acids are supplemented [39], which also is the likely reason that higher osmotic tolerance is observed when *E. coli* is grown in rich media rather than in minimal media without amino acids. However, higher concentrations of some amino acids (e.g., serine, cysteine, threonine, isoleucine, and leucine) reduced growth, likely due to feedback inhibition. Interestingly, the addition of 100 µg mL^−1^ methionine appears to result in a faster accumulation of biomass (based on the higher biomass concentration at 24 h), but a 39% lower final biomass (*p*-value < 0.001) after 48 h in strain EJW3. However, the addition of 100 µg mL^−1^ methionine led to a 72% lower final biomass (*p*-value < 0.001) in BW25113 after 48 h, which suggests a higher feedback inhibition of methionine in strain BW25113 compared with EJW3 (Figure 3). The supplementation of methionine has been reported to improve the tolerance to NaCl in *Saccharomyces cerevisiae* [40], but has not been reported in *E. coli*. Our results suggest potential synergy between the *rpoC* mutation and methionine supplementation on osmotic tolerance in *E. coli*. The supplementation with 100 µg mL^−1^ and 1000 µg mL^−1^ of proline (Figure 4) or with 1000 µg mL^−1^ of alanine (Figure 5) resulted in significant growth inhibition in EJW3, but were beneficial to BW25113, in the presence of osmotic stress. This led us to hypothesize that EJW3 may produce more proline and alanine, making it more sensitive to additional supplementation of these two amino acids, compared with BW25113. Proline has been reported as an osmoprotectant in *E. coli* [38,41] and other microorganisms [42]. Thus, the overproduction of proline may be one of the causes that contribute to the higher osmotic tolerance in EJW3.

### 3.3. Metabolite Analysis

In order to test our hypothesis that *rpoC* K370_A396dup mutation led to an overproduction of some amino acids such as proline and alanine, and to identify other potential effects of the mutation on the production of other metabolites, extracellular and intracellular metabolites of BW25113 and EJW3 during late exponential phase (OD_600_ about 0.7–1.0) in the presence and absence of osmotic stress were analyzed. The results are shown in Table 4, Table 5, Tables S7 and S8. The results showed that strain EJW3 produced ~22% more intracellular (*p*-value < 0.001) and ~50% more extracellular (*p*-value < 0.001) proline than BW25113 in the absence of hyperosmotic stress, while the exposure to 0.6 M NaCl led to increased intracellular amount of proline in both strains and extracellular amount of proline produced by BW25113. Prior study has shown the overproduction of proline to confer enhanced osmotic stress tolerance [43]. Since the inoculum used in our study were prepared without the addition of excess NaCl, the EJW3 culture likely contained higher initial intracellular proline than BW25113, which may result in early protection to EJW3 from the inhibition of 0.6 M NaCl. Glutamic acid has been found to accumulate in osmotically stressed cells and serves as an osmoprotectant [15]. Consistent with prior work, results from our experiment also showed an increase in glutamic acid production under osmotic stress in both BW25113 and EJW3 strains. However, EJW3 appears to accumulate more glutamic acid intracellularly, as the intracellular level of glutamic acid was ~24% higher (*p*-value = 0.006), while the extracellular concentration was ~62% lower (*p*-value < 0.001) in EJW3 compared with BW25113. This suggests that EJW3 may be better at accumulating glutamic acid intracellularly under osmotic stress due to reduced export of the amino acid into the bulk medium. A similar trend was observed with arginine levels, with both strains producing more arginine in the presence of osmotic stress, and EJW3 maintaining ~56% higher intracellular arginine concentration (*p*-value < 0.001) compared with the wild-type (Table 5). It has also been reported that cold osmotic shock reduced the ability of *E. coli* to accumulate arginine [44]. The ability of EJW3 to produce and accumulate more arginine than BW25113 may also contribute to its higher osmotic tolerance. Alanine is known to be an important osmolyte in many organisms [45], but thus far has not been reported to serve as an osmolyte in *E. coli*. Our results showed that the production of alanine decreased under osmotic stress in both strains, but the intracellular concentration was ~86% higher in EJW3 than in BW25113 (*p*-value = 0.001). Though the production of alanine did not increase in response to osmotic stress, the data suggests that biosynthesis of this amino acid was not significantly inhibited in EJW3 in the presence of osmotic stress compared with BW25113. In addition to known amino acids that are perturbed by osmotic stress in bacteria, our results showed that the production of methionine, tyrosine, tryptophan, phenylalanine and isoleucine were also perturbed by osmotic stress and the *rpoC* mutation (Tables S7 and S8). 

Trehalose is one of the most well-known osmoprotectants. Prior reports showed increased production of trehalose in response to osmotic stress to prevent water loss and to maintain the intracellular pressure of the cells [15,18]. We found that the intracellular concentration of trehalose under osmotic stress to be ~37% higher in EJW3 than BW25113 (*p*-value = 0.005), which may be another reason for the higher osmotic tolerance conferred by the *rpoC* mutation.

In addition to known osmoprotectants and amino acids, we also compared the intracellular and extracellular levels of organic acids between the two strains. The results showed a 30%–40% higher extracellular acetic acid concentration in EJW3 cultures (*p*-value < 0.001) with or without osmotic stress challenge. However, EJW3 showed a ~40% lower intracellular acetic acid concentration in the absence of (*p*-value < 0.001), and a similar level of intracellular acetic acid concentration in the presence of (*p*-value = 0.137) hyperosmotic stress. Taking into account the relative total volume of extracellular versus intracellular metabolites collected (a ratio of 100:1.5), the total amount of acetic acid produced by EJW3 (370 ± 20 µg mL^−1^ OD_600_^−1^) was about 30% higher (*p*-value < 0.001) than that of BW25113 (290 ± 20 µg mL^−1^ OD_600_^−1^). During growth on glucose as carbon source, the acetate secretion of *E. coli* has been shown to depend on growth rate [46,47], thus the higher level of extracellular acetic acid in strain EJW3 may not be a direct consequence of the *rpoC* mutation but a side effect of the higher growth rate of the *rpoC* mutant under osmotic stress. However, it is possible that the higher acetic acid production may also contribute to the higher osmotic tolerance of EJW3.

### 3.4. Effect of Acetic Acid on Osmotic Stress Tolerance

**Figure 6:**
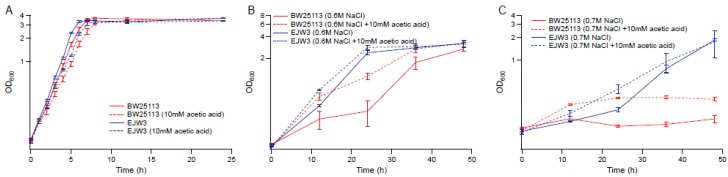
Effect of acetic acid on osmotic tolerance. (**A**) M9 only. (**B**) M9 supplemented with 0.6 M NaCl. (**C**) M9 supplemented with 0.7 M NaCl. Error bars are standard deviations.

To determine the role acetic acid plays on osmotic tolerance, we compared the growth kinetics of BW25113 and EJW3 in the presence of 10 mM acetic acid in M9 with or without the supplementation of 0.6 M NaCl. In the absence of osmotic stress, the addition of 10 mM acetic acid showed no significant impact on the growth of either strain (Figure 6A). However, the addition of acetic acid significantly improved the growth of BW25113 but only had a moderate impact on the growth of EJW3 in the presence of NaCl challenge (Figure 6B). Under higher osmotic stress with 0.7 M NaCl, acetic acid addition still had a benefit on the growth of BW25113, and showed a moderate benefit on the growth of EJW3 (Figure 6C). Prior work has shown that moderate concentration of NaCl can protect *E. coli* from acetic acid toxicity [48]; here we show that the cross tolerance is reciprocal, that moderate concentrations of acetic acid can also protect *E. coli* from NaCl stress. Interestingly, a statistically significant overlap (*p* < 10^−20^, Fisher’s exact test) between metabolites level changes in organic acid and osmotolerant *E. coli* mutants has been detected using an updated version of the Resistome combined with a recent genome-wide survey of genotype–metabolite relationships [49,50], providing additional evidence that the improved osmotic tolerance of EJW3 may also result from the higher production of acetic acid.

### 3.5. Cell Membrane Damage Analysis

**Figure 7:**
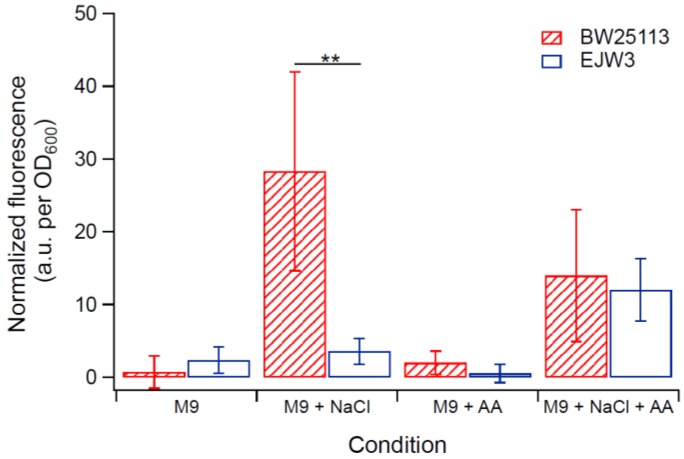
Membrane integrity assay using PI staining of exponential growing cells. NaCl: 0.7 M NaCl. AA: 10 mM acetic acid. ** Statistically significantly different using two-tailed student’s *t*-test (*p*-value < 0.005). Error bars are standard deviations.

As osmotic stress perturbs the membrane, propidium iodide (PI) assay was used to assess any differences in membrane perturbation between BW25113 and EJW3 in the presence of hyperosmotic stress in different growth phases. Propidium iodide is a fluorescent molecule that normally cannot penetrate into cells with intact membrane [51,52]; however, if the membrane integrity is compromised, the uptake of PI increases, and can be used to assess relative levels of membrane perturbation. In order to detect differences within a short-term challenge, a higher hyperosmotic stress with 0.7 M NaCl was used in this test. The membrane integrity of both strains was assessed after a 30-min exposure to (1) M9 only, (2) M9 supplemented with 0.7 M NaCl, (3) M9 supplemented with 10 mM acetic acid, and (4) M9 supplemented with 0.7 M NaCl and 10 mM acetic acid. Cells in either lag phase (OD_600_ ~ 0.1), exponential phase (OD_600_ ~ 0.8) or stationary phase (OD_600_ ~ 3.0) were tested. Low levels of PI staining were observed in both strains in lag phase and stationary phase cells with or without NaCl challenge (Figure S2), suggesting no major membrane perturbation by NaCl challenge during these non-growth phases. However, in exponential growing cells, BW25113 showed significantly higher PI staining than EJW3 (Figure 7) under 0.7 M NaCl challenge, suggesting a lower level of membrane perturbation in the *rpoC* mutant under hyperosmotic stress. While our data showed that addition of 10 mM acetic acid improved growth of BW25113 in hyperosmotic stress (Figure 6), PI staining data revealed that the addition of acetic acid conferred no benefit to the hyperosmotic stress induced membrane perturbation in either strain, which suggests that the benefit of acetic acid to osmotic tolerance is not related to membrane integrity. 

### 3.6. Transcriptional Profile Analysis

RpoC is the β’ subunit of the RNA polymerase complex, and plays roles in promoter recognition, sigma factor binding and ion chelation [53,54,55]. The *rpoC* K370_A396dup mutation is located in domain 2 (amino acid residues 344–486) of the RpoC subunit [56], close to the clamp (amino acid residues 14–342) and the NADFDGD (amino acid residues 458–464) motif which is involved in Mg2+ binding [55,57]; thus, this mutation in *rpoC* may impact transcriptional initiation and elongation [58,59]. Furthermore, the 3-D structure of the RNA polymerase places the K370_A396dup mutation in close proximity to the proposed binding site of guanosine tetraphosphate (ppGpp) (amino acid residues 362, 417) [60,61,62], which is a global regulator involved in stringent response [63]. Thus, the *rpoC* K370_A396dup mutation is expected to have global impact on the transcriptional regulation of the cell; therefore, the transcriptional differences in BW25113 and EJW3 can potentially be used to identify new molecular mechanisms of osmotolerance in *E. coli*. Thus, we compared the transcriptional profiles between EJW3 and BW25113 in M9 with or without 0.6 M NaCl challenge to identify any transcriptional regulatory differences that may lead to new findings on molecular mechanisms for osmotic tolerance. The transcriptional data showed that most genes upregulated in strain EJW3 under osmotic stress are related with amino acids metabolism, which corroborated with our data from amino acid supplementation and metabolite analysis; and the genes downregulated are related with membrane composition and transporters. 

**Figure 8:**
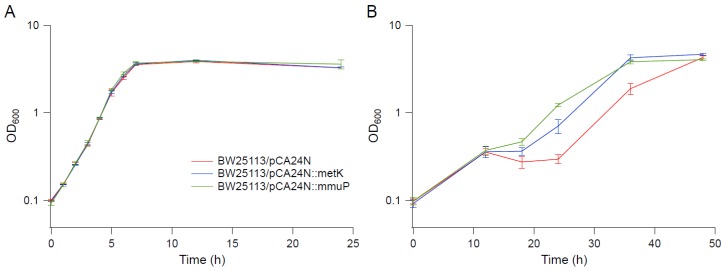
Growth kinetics of overexpression strains in BW25113 background. (**A**) M9. (**B**) M9 supplemented with 0.55 M NaCl. Error bars are standard deviations.

**Figure 9:**
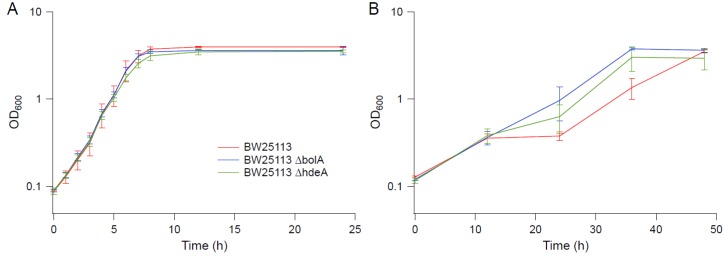
Growth kinetics of knockout strains in BW25113 background. (**A**) M9. (**B**) M9 supplemented with 0.6 M NaCl. Error bars are standard deviations.

Several genes upregulated in strain EJW3 under osmotic stress were chosen for further validation for their role in osmotic tolerance in the *rpoC* K370_A396dup background. The selected genes (Table S9) are involved in amino acids metabolism and were chosen based on results from the amino acid supplementation and metabolite analysis. These genes were overexpressed in BW25113 by using clones from the ASKA collection [35], and the growth kinetics of the overexpression strains were compared against the control expressing the empty plasmid in M9 with or without NaCl supplementation. To avoid potential toxicity associated with high level gene expression, basal level expression (without Isopropyl β-D-1-thiogalactopyranoside (IPTG) induction) and a lower osmotic stress (0.55 M NaCl) were used. Among the genes tested, only the overexpression of *metK* and *mmuP* significantly improved the performance of BW25113 under osmotic stress. The overexpression of *metK* and *mmuP* did not impact the growth of BW25113 in the absence of hyperosmotic stress, but rather conferred a benefit in the presence of 0.55 M NaCl (Figure 8). This suggests that the osmotic tolerance conferred by the *rpoC* mutation in BW25113 is partially due to the upregulation of *metK* and *mmuP*. *MetK* encodes the methionine adenosyltransferase that catalyzes the formation of the S-adenosylmethionine (AdoMet) [64], which is involved in many biological reactions. It plays important roles as donors of methyl, sulfur, and aminopropyl groups, thus is frequently involved in the regulation of gene expression, including genes involved in methionine metabolism [65,66,67,68], but was not known to be related to osmotolerance. *MmuP* is a transporter of S-methylmethionine, which can be used by *E. coli* as a source of methionine when externally provided [69]. S-methylmethionine is widely produced by plants and can be used as an osmoprotectants in plants [70], but it has not been reported as an osmoprotecant in *E. coli*. As *E. coli* is not known to produce S-methylmethionine, nor did we supplement S-methylmethionine into the media, in this case, the impact of *mmuP* on osmotic tolerance is not obvious. Stress response genes *bolA* and *hdeA* are downregulated in the BW25113 *rpoC* mutant, and the deletion of *bolA* and *hdeA* improved the growth of BW25113 in the presence of 0.6 M NaCl (Figure 9). BolA is a transcriptional regulator involved in regulating cell morphology related genes, and is known to be involved in general stress response and has been reported to be overexpressed under osmotic stress [71]; however, the mechanism of how it is involved in osmotic tolerance remains unclear. HdeA is a periplasmic acid stress chaperone which plays a role in resistance to low pH [72,73], and it is not known to be related to osmotic tolerance. 

As stated previously, the K370_A396dup mutation is close to the proposed ppGpp binding site on RpoC [60,61,62]. PpGpp is known to be involved in stringent response upon nutrition starvation or other stress conditions, and has global impact on gene expression [63,74]. It regulates transcriptional initiation directly by binding to an interface between the β′ and ω subunit of the RNA polymerase, or indirectly by altering sigma factor availability, and it also indirectly affects translation and DNA replication [74,75]. It has recently been reported that the supplementation of serine hydroxamate (SHX), which induces production of ppGpp, increased osmotic tolerance of ppGpp-proficient *E. coli*, which was not observed in ppGpp-deficient cells, demonstrating that ppGpp is involved in osmotic tolerance [76]. Furthermore, an RpoC Δ312–315 mutation was previously found to suppress the ppGpp deficiency phenotypes [77]. Since the *rpoC* K370_A396dup mutation falls outside the amino acid residues known to impact RpoC interaction with ppGpp, any relationship between the *rpoC* K370_A396dup mutation and ppGpp remains to be investigated. 

### 3.7. Impact of the rpoC Mutation in MG1655

**Figure 10:**
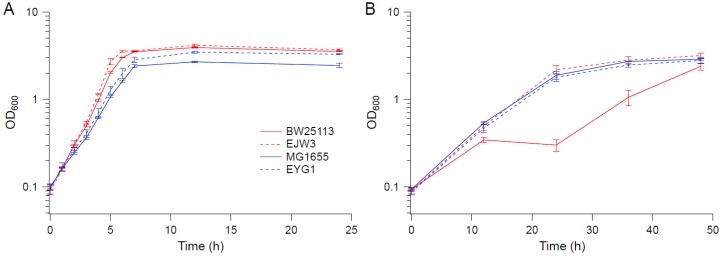
Growth kinetics of MG1655, EYG1, BW25113 and EJW3. (**A**) M9 (**B**) M9 supplemented with 0.6 M NaCl. Error bars are standard deviations.

To determine whether the *rpoC* mutation exhibits a similar benefit to osmotic stress tolerance in other *E. coli* K-12 strains, the *rpoC* mutation was reconstructed in MG1655 to generate strain EYG1. The osmotic tolerance of MG1655 and EYG1 were assessed in M9 with or without challenge with 0.6 M NaCl (shown in Figure 10A,B, respectively). In contrast to BW25113, the *rpoC* K370_A396dup mutation did not confer a benefit to MG1655 under osmotic stress. Though the growth of both MG1655 and EYG1 were slower than BW25113 in M9 in the absence of hyperosmotic stress (Figure 10A), their growths were nearly identical to EJW3 under osmotic stress (Figure 10B), suggesting that the inherent tolerance of MG1655 to NaCl was higher than BW25113, and led us to use a higher NaCl concentration with subsequent assays with MG1655. 

**Figure 11:**
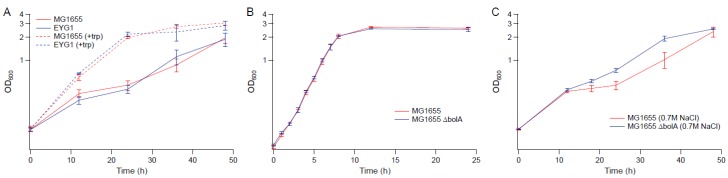
Growth kinetics of MG1655, EYG1 and the knockout strains in MG1655 background. (**A**) M9 supplemented with 0.7 M NaCl with and without 50 µg mL^−1^ tryptophan (**B**) M9 (**C**) M9 supplemented with 0.7 M NaCl. Error bars are standard deviations.

As we found tryptophan supplementations to benefit BW25113 in the presence of hyperosmotic challenge, we also assessed its potential benefit in MG1655. In the presence of 0.7 M NaCl, addition of tryptophan to the medium increased the osmotic tolerance of MG1655 and EYG1 (Figure 11A). The deletion of *bolA* also improved the growth of MG1655 under osmotic stress with 0.7 M NaCl (Figure 11B,C), which suggests that although the impact of the *rpoC* mutation on osmotic tolerance appears to be strain specific, some of the findings based on BW25113 also applies to MG1655. 

We also compared the transcriptional profiles between MG1655 and EYG1 (MG1655 background) in M9 with or without 0.6 M NaCl challenge to identify any transcriptional regulatory differences caused by the *rpoC* mutation between MG1655 and BW25113 background. The microarray data showed that the *rpoC* mutation caused significantly different transcriptional perturbation between these two strain backgrounds, with few similarities shared between strains and conditions. As mentioned previously, most genes upregulated in strain EJW3 under osmotic stress are related with amino acids metabolism; however, in EYG1 (MG1655 background), most amino acids metabolism and transport related genes perturbed by the *rpoC* mutation were downregulated (Table S10). This difference between strains EJW3 and EYG1 likely explains the different physiological impacts of the *rpoC* mutation on osmotic tolerance between BW25113 and MG1655. 

## 4. Conclusions

In this work, we confirmed that the in-frame, 84 bp duplication in *rpoC* (K370_A396dup) does indeed contribute to osmotic tolerance of *E. coli*. The *rpoC* K370_A396dup mutation was reconstructed in the BW25113 background (EJW3), and the *rpoC* mutant performed much better than the wild-type strain under osmotic stress. From individual amino acid supplementation studies, we found several additional amino acids (e.g., tryptophan, phenylalanine, etc.) to play a role in improving osmotic tolerance in *E. coli*. Results from metabolite analysis revealed differences in intracellular and extracellular metabolites between the wild-type and *rpoC* mutant, suggesting alterations in metabolism may be partially responsible for the enhanced hyperosmotic tolerance in the mutant. Compared with the wild-type strain, EJW3 produced and accumulated more known osmoprotectants (trehalose, proline, and glutamic acid) in response to hyperosmotic stress. In addition to known osmolytes, EJW3 also produce approximately 30% more acetic acid than BW25113. Subsequent supplementation studies confirmed that the addition of moderate concentration of acetic acid helps to improve the performance of BW25113 under hyperosmotic stress; which supported the theory that the higher production of acetic acid is likely related with the osmotic tolerance conferred by the *rpoC* (K370_A396dup) mutation. Membrane damage analysis using PI staining showed a lower effect on membrane integrity in the presence of hyperosmotic stress in *rpoC* mutants that are actively growing (in exponential growth phase). Transcriptional analysis demonstrated that the *rpoC* mutation indeed impacted relative transcript abundance of genes related with amino acids metabolism, including *metK* and *mmuP*. When overexpressed in BW25113, *metK* and *mmuP* conferred enhanced osmotic tolerance, providing further validation that methionine metabolism is involved in enhanced hyperosmotic tolerance in the *rpoC* mutant. Deletion of stress response genes *bolA* and *hdeA* also improved growth of BW25113 under osmotic stress, and the benefit of *bolA* deletion on osmotic tolerance also applies to MG1655. Other genes perturbed by the *rpoC* mutation in BW25113 and the synergistic effects of those genes remain to be investigated. In conclusion, the work demonstrated that the impacts of this particular *rpoC* mutation on *E. coli* metabolism and membrane integrity related with osmotic tolerance, and although the impacts of *rpoC* mutation appeared to be strain dependent, some of the findings in BW25113 also apply to another K-12 strain. Results from this work can help to identify targets for metabolic engineering of *E. coli* for enhanced tolerance to alternative feedstocks and water sources.

## Figures and Tables

**Table 1:** List of strains.

Strains	Description/Genotype	Source
BW25113	F-, Δ*(araD-araB)567*, *lacZ4787(del)::rrnB-3*, *λ-*, *rph-1*, Δ*(rhaD-rhaB)568*, *hsdR514*	CGSC
MG655	F-, *λ-*, *rph-1*	CGSC
Hfr-2×SFX-	BW25113 Δ*mbhA::oriT*, Δ*hyfC::oriT*, *trp::F[ΔtraST]*, (*gen^R^*), parental strain of G3	[24]
G3	Evolved mutant of Hfr-2×SFX- containing *rpoC* K370_A396dup mutation	[24]
JW3929	BW25113 Δ*argE744::kan*	[25]
JW1253	BW25113 Δ*trpB769::kan*	[25]
EJW1	G3 Δ*argE744::kan*	This study
EJW2	BW25113 Δ*argE744::kan*, *rpoC* K370_A396dup	This study
EJW3	BW25113 *rpoC* K370_A396dup	This study
EJW4	BW25113 *rpoC* K370_A396dup, Δ*trpB769::kan*	This study
EYG1	MG1655 *rpoC* K370_A396dup	This study

**Table 2:** Growth in micro-aerobic condition in M9 supplemented with 0.8 M NaCl.

Strains	0 h	24 h	48 h	72 h
BW25113	−	+	+	+
EJW3	−	+	+++	++++
JW1253 *	−	+	++	+
EJW4 *	−	++	++++	++++
Hfr-2×SFX- *	−	+	+	+
G3 *	−	+++	++++	+++

* Tryptophan (50 µg mL^−1^) was supplemented; − OD_600_ < 0.1, + 0.1 < OD_600_ < 0.5, ++ 0.5 < OD_600_ < 1.0, +++ 1.0 < OD_600_ < 2.0, ++++ OD_600_ > 2.0.

**Table 3:** Growth in aerobic condition in M9 supplemented with 0.75 M NaCl.

Strains	0 h	24 h	48 h	72 h
BW25113	−	+	+	+
EJW3	−	+	+++	++++
JW1253 *	−	+	++	+
EJW4 *	−	++	++++	++++
Hfr-2×SFX- *	−	+	+	+
G3 *	−	+++	++++	+++

* Tryptophan (50 µg mL^−1^) was supplemented; − OD_600_ < 0.1, + 0.1 < OD_600_ < 0.5, ++ 0.5 < OD_600_ < 1.0, +++ 1.0 < OD_600_ < 2.0, ++++ OD_600_ > 2.0.

**Table 4:** Quantification of intracellular and extracellular metabolites in M9.

	Metabolite	BW25113	EJW3	*p*-Value
Intracellular	Trehalose (µg mL^−1^ OD_600_^−1^)	-	-	-
	Acetic acid (µg mL^−1^ OD_600_^−1^)	650 ± 90	***390 ± 80***	0.000 *
	Glu (µg mL^−1^ OD_600_^−1^)	187.72 ± 35.94	212.37 ± 27.2	0.210
	Ala (µg mL^−1^ OD_600_^−1^)	81.18 ± 16.55	83.71 ± 3.15	0.721
	Arg (µg mL^−1^ OD_600_^−1^)	-	4.78 ± 7.41	0.145
	Pro (µg mL^−1^ OD_600_^−1^)	61.64 ± 6.12	**75.06 ± 1.95**	0.000 *
Extracellular	Trehalose (µg mL^−1^ OD_600_^−1^)	-	-	-
	Acetic acid (µg mL^−1^ OD_600_^−1^)	280 ± 20	**370 ± 10**	0.000 *
	Glu (µg mL^−1^ OD_600_^−1^)	-	6.42 ± 12.41	0.234
	Ala (µg mL^−1^ OD_600_^−1^)	10.86 ± 6.33	***1.93 ± 4.72***	0.020 *
	Arg (µg mL^−1^ OD_600_^−1^)	2.95 ± 7.21	-	0.341
	Pro (µg mL^−1^ OD_600_^−1^)	516.13 ± 35.09	**776.02 ± 117.02**	0.000 *

- Not detectible. * Statistically significantly different between BW25113 and EJW3 (*p*-values < 0.05). Values bolded are significantly higher in EJW3. Values bolded and italicized are significantly lower in EJW3. *p*-values are calculated by using a two-tailed student’s *t*-test with six biological replicates.

**Table 5:** Quantification of intracellular and extracellular metabolites in M9 supplemented with 0.6 M NaCl.

	Metabolite	BW25113	EJW3	*p*-Value
Intracellular	Trehalose (µg mL^−1^ OD_600_^−1^)	1190 ± 190	**1630 ± 220**	0.005 *
	Acetic acid (µg mL^−1^ OD_600_^−1^)	270 ± 140	370 ± 50	0.137
	Glu (µg mL^−1^ OD_600_^−1^)	565.50 ± 44.39	**698.75 ± 82.47**	0.006 *
	Ala (µg mL^−1^ OD_600_^−1^)	38.49 ± 9.03	**71.58 ± 13.51**	0.001 *
	Arg (µg mL^−1^ OD_600_^−1^)	28.79 ± 2.73	**44.87 ± 6.42**	0.000 *
	Pro (µg mL^−1^ OD_600_^−1^)	94.31 ± 6.14	107.31 ± 18.37	0.131
Extracellular	Trehalose (µg mL^−1^ OD_600_^−1^)	-	-	-
	Acetic acid (µg mL^−1^ OD_600_^−1^)	260 ± 20	**360 ± 20**	0.000 *
	Glu (µg mL^−1^ OD_600_^−1^)	234.82 ± 15.44	***88.05 ± 19.38***	0.000 *
	Ala (µg mL^−1^ OD_600_^−1^)	-	-	-
	Arg (µg mL^−1^ OD_600_^−1^)	-	7.90 ± 12.34	0.148
	Pro (µg mL^−1^ OD_600_^−1^)	747.75 ± 120.88	728.90 ± 156.43	0.820

- Not detectible. * Statistically significantly different between BW25113 and EJW3 (*p*-values < 0.05). Values bolded are significantly higher in EJW3. Values bolded and italicized are significantly lower in EJW3. *p*-values are calculated by using a two-tailed student’s *t*-test with six biological replicates.

## Supplementary Materials

The supplementary materials are available online at http://www.mdpi.com/2306-5354/4/3/61/s1, Table S1: Reagents used in analysis of amino acids, Table S2: Injection program, Table S3: Mobile phase gradients, Table S4: Growth in micro-aerobic condition in M9 supplemented with 0.9 M NaCl, Table S5: Growth in aerobic condition in M9 supplemented with 0.8 M NaCl , Table S6: Cell density (OD_600_) in M9 supplemented with 0.65 M NaCl and amino acids, Table S7: Quantification of intracellular and extracellular metabolites in M9, Table S8: Quantification of intracellular and extracellular metabolites in M9 supplemented with 0.6 M NaCl, Table S9: Upregulated genes selected for validation for their roles in osmotic tolerance, Table S10: Gene ontology analysis in M9 supplemented with 0.6 M NaCl, Figure. S1: Light microscopy of cells in the presence or absence of hyperosmotic stress, Figure. S2: Membrane integrity assay using PI staining. (A) lag phase cells. (B) Early stationary phase cells. (C) Late stationary phase cells. NaCl: 0.7 M NaCl. AA: 10 mM acetic acid. Error bars are standard deviations.
