# Supplementary material for: Insights on Osmotic Tolerance Mechanisms in Escherichia coli Gained from an rpoC Mutation
Source: Bioengineering (Basel). 2017 Jun 28;4(3):61. doi: 10.3390/bioengineering4030061 (PMC5615307; doi:10.3390/bioengineering4030061)
Supplement: Supplementary file 1 [file bioengineering-04-00061-s001.pdf]

bioengineering

# Supplementary materials

## 1. Supplementary methods

### *1.1. Metabolites extraction*

Single colonies were inoculated in 10 mL M9 media in test tubes, incubate at 37 °C aerobically until $OD_{600}$ reached ~1.0. The cells were centrifuged at 2,800× g for 10 minutes, supernatants were removed and the pellets were resuspended in M9 at an $OD_{600}$ of ~10.0. Then 500 µL samples were inoculated into 50 mL M9 or M9 supplemented with 0.6 M NaCl in 250 mL screw-capped flasks at an $OD_{600}$ of ~0.1, and incubated at 37 °C with shaking (275 rpm). Two technical replicates were made for each sample. When $OD_{600}$ of samples reached exponential phase ($OD_{600}$ ~0.7 - 1), the two technical replicates with a total volume of 100 mL were combined and cells were harvested by centrifugation at 9,000× g, 1 mL supernatant with extracellular metabolites was filtered using 0.2 µm syringe filter (VWR, Radnor, PA USA) and kept at -20 °C. The remaining supernatants were removed completely. The cell pellets were washed with 5 mL fresh media, and then the supernatants were removed completely. Intracellular metabolites were extracted by suspending the cell pellets in 500 µL -20 °C intracellular metabolites extraction buffer (acetonitrile: methanol: water (40:40:20)) [1] and incubated at -20 °C for 30 minutes. Then samples were centrifuged (9,000× g) at 4 °C for 5 minutes, and the supernatant was kept at -20 °C. This extraction step was repeated for another two times using 400 µL and 300 µL intracellular metabolites extraction buffer (acetonitrile: methanol: water (40:40:20)) respectively with 15 minutes incubation at -20 °C and then centrifuged (9,000× g) at 4 °C for 5 minutes. The supernatants from the three extraction steps were combined. Then 500 µL water was combined with l mL extracted intracellular metabolites, filtered using 0.2 µm syringe filter (VWR, Radnor, PA USA) and kept at -20°C.

### *1.2. HPLC analysis of free amino acids*

Free amino acids were analyzed using modified Agilent amino acid analysis method [2]. We used high-performance liquid chromatography (HPLC; Agilent Technologies, 1260 Infinity, Santa Clara, CA) and Cogent Bidentate C18™ (4 µm, 100 Å. Dimensions: 4.6 mm i.d. x 150 mm) column (MicroSolv Technology, Eatontown, NJ USA) at 40 °C. Flow rate of mobile phase was 0.6 mL $min^{-1}$. The mobile phase and reagents are listed in Table S1. Injection program and mobile phase gradients are listed in Table S2 and Table S3. The detection wavelength was 338 nm from 0 - 18 min, and 262 nm from 18 - 30 min.

## 2. Supplementary Tables

**Table S1.** Reagents used in analysis of amino acids.

| Reagents | Composition |
|---|---|
| Mobile phase A | 10 mM $Na_2HPO_4$: 10 mM $Na_2B_4O_7$: 5 mM $NaN_3$, pH 8.2 |
| Mobile phase B | acetonitrile: methanol: water (45:45:10, v: v: v) |
| Borate buffers | 0.4 M in water, pH 10.2 |
| FMOC | 2.5 mg $mL^{-1}$ in acetonitrile |
| OPA | 10 mg $mL^{-1}$ Phthaldialdehuyde and 3-Mercaptopropionic acids in 0.4 M borate buffer |

**Table S2.** Injection program.

| Steps | Injection program |
|---|---|
| 1 | Draw 12.5 μL from borate vial. |
| 2 | Draw 5 μL from sample vial. |
| 3 | Mix 17.5 μL from air 5 times. |
| 4 | Wait 0.2 min. |
| 5 | Draw 2.5 μL from OPA vial. |
| 6 | Mix 20 μL from air 10 times default speed. |
| 7 | Draw 2 μL from FMOC vial. |
| 8 | Mix 22 μL from air 10 times default speed. |
| 9 | Inject. |
| 10 | Wait 0.1 min. |
| 11 | Valve bypass. |

**Table S3.** Mobile phase gradients.

| Time (min) | Mobile phase A (%) | Mobile phase B (%) |
|---|---|---|
| 0 | 98 | 2 |
| 0.5 | 98 | 2 |
| 20 | 43 | 57 |
| 20.1 | 0 | 100 |
| 23.5 | 0 | 100 |
| 23.6 | 98 | 2 |
| 25 | 98 | 2 |
| 35 | 98 | 2 |

**Table S4.** Growth in micro-aerobic condition in M9 supplemented with 0.9 M NaCl.

| Strains | 0 h | 24 h | 48 h | 72 h |
|---|---|---|---|---|
| BW25113 | - | + | - | - |
| EJW3 | - | + | - | - |
| JW1253* | - | + | + | + |
| EJW4* | - | + | + | + |
| Hfr-2×SFX-* | - | + | + | + |
| G3* | - | + | + | + |

* Tryptophan (50 μg $mL^{-1}$) was supplemented

- $OD_{600} < 0.1$, + $0.1 < OD_{600} < 0.5$, ++ $0.5 < OD_{600} < 1.0$, +++ $1.0 < OD_{600} < 2.0$, ++++ $OD_{600} > 2.0$

**Table S5.** Growth in aerobic condition in M9 supplemented with 0.8 M NaCl.

| Strains | 0 h | 24 h | 48 h | 72 h |
|---|---|---|---|---|
| BW25113 | - | - | - | - |
| EJW3 | - | + | - | - |
| JW1253* | - | + | + | + |
| EJW4* | - | + | + | + |
| Hfr-2×SFX-* | - | + | - | - |
| G3* | - | + | + | + |

* Tryptophan (50 μg $mL^{-1}$) was supplemented

- $OD_{600} < 0.1$, + $0.1 < OD_{600} < 0.5$, ++ $0.5 < OD_{600} < 1.0$, +++ $1.0 < OD_{600} < 2.0$, ++++ $OD_{600} > 2.0$

**Table S6.** Cell density ($OD_{600}$) in M9 supplemented with 0.65 M NaCl and amino acids.

| Amino acid | Concentration (μg mL⁻¹) | 24h | | 48h | |
|---|---|---|---|---|---|
| | | BW25113 | EJW3 | BW25113 | EJW3 |
| Control[a] | 0 | 0.22 ± 0.09 | 0.70 ± 0.31 | 0.90 ± 0.45 | 3.03 ± 0.30 |
| Ala | 10 | 0.34 ± 0.09 | 1.19 ± 0.44 | 0.80 ± 0.44 | 2.92 ± 0.29 |
| | 100 | **1.77 ± 0.58*** | **2.78 ± 0.30*** | **2.20 ± 0.10*** | 3.11 ± 0.15 |
| | 1000 | 0.93 ± 0.33 | ***0.22 ± 0.10**** | **4.13 ± 0.47*** | ***0.45 ± 0.26**** |
| Arg | 10 | ***0.14 ± 0.01**** | 1.76 ± 0.53 | 0.71 ± 0.16 | 3.57 ± 0.38 |
| | 100 | **1.70 ± 0.44*** | **2.84 ± 0.17*** | **2.90 ± 0.33*** | **3.77 ± 0.20*** |
| | 1000 | **2.40 ± 0.26*** | **2.69 ± 0.26*** | **2.93 ± 0.58*** | 3.23 ± 0.29 |
| Asn | 10 | **0.41 ± 0.02*** | **1.90 ± 0.42*** | **2.63 ± 0.14*** | 3.49 ± 0.25 |
| | 100 | **3.16 ± 0.30*** | **3.04 ± 0.19*** | **3.30 ± 0.10*** | **4.02 ± 0.15*** |
| | 1000 | **3.04 ± 0.46*** | **3.45 ± 0.29*** | **3.44 ± 0.22*** | 4.12 ± 0.52 |
| Asp | 10 | 0.33 ± 0.08 | 0.60 ± 0.12 | 1.02 ± 0.46 | ***2.77 ± 0.10**** |
| | 100 | **1.82 ± 0.29*** | **2.77 ± 0.21*** | **2.50 ± 0.09*** | 3.08 ± 0.32 |
| | 1000 | **2.21 ± 0.52*** | **3.13 ± 0.09*** | **2.27 ± 0.31*** | **3.65 ± 0.22*** |
| Cys | 10 | **2.68 ± 0.25*** | **2.95 ± 0.25*** | **3.10 ± 0.24*** | **4.04 ± 0.25*** |
| | 100 | **0.78 ± 0.22*** | **1.60 ± 0.12*** | **2.68 ± 0.05*** | ***2.33 ± 0.04**** |
| | 1000 | ***0.10 ± 0.01**** | ***0.09 ± 0.01**** | 1.90 ± 0.78 | 1.05 ± 1.47 |
| Glu | 10 | 0.19 ± 0.03 | **1.16 ± 0.10*** | **1.46 ± 0.12*** | 2.84 ± 0.19 |
| | 100 | **1.88 ± 0.44*** | **2.64 ± 0.11*** | **2.56 ± 0.18*** | 3.04 ± 0.19 |
| | 1000 | **2.48 ± 0.39*** | **2.74 ± 0.12*** | **3.33 ± 0.51*** | 3.35 ± 0.31 |
| Gln | 10 | 0.29 ± 0.08 | 0.77 ± 0.16 | 0.98 ± 0.38 | 3.01 ± 0.29 |
| | 100 | **0.94 ± 0.10*** | **2.76 ± 0.06*** | **2.69 ± 0.13*** | **3.30 ± 0.14*** |
| | 1000 | **2.75 ± 0.41*** | **3.19 ± 0.52*** | **3.09 ± 0.22*** | **3.86 ± 0.35*** |
| Gly | 10 | **0.40 ± 0.01*** | **1.88 ± 0.05*** | **1.85 ± 0.14*** | 3.37 ± 0.19 |
| | 100 | **2.01 ± 0.14*** | **3.17 ± 0.23*** | **2.79 ± 0.07*** | **3.83 ± 0.05*** |
| | 1000 | 0.50 ± 0.13 | **2.17 ± 0.36*** | ***0.28 ± 0.07**** | **3.52 ± 0.09*** |
| His | 10 | **2.38 ± 0.31*** | **3.05 ± 0.20*** | **3.27 ± 0.18*** | **4.15 ± 0.18*** |
| | 100 | **2.48 ± 0.23*** | **2.80 ± 0.15*** | **3.24 ± 0.07*** | **3.85 ± 0.26*** |
| | 1000 | **2.63 ± 0.21*** | **2.96 ± 0.10*** | **3.12 ± 0.30*** | **3.54 ± 0.12*** |
| Ile | 10 | 0.23 ± 0.04 | **1.72 ± 0.14*** | **1.29 ± 0.13*** | 3.15 ± 0.23 |
| | 100 | **2.04 ± 0.07*** | **2.97 ± 0.07*** | **1.81 ± 0.16*** | **3.69 ± 0.16*** |
| | 1000 | **0.39 ± 0.05*** | ***0.21 ± 0.02**** | ***0.46 ± 0.06**** | ***0.22 ± 0.02**** |
| Leu | 10 | 0.62 ± 0.19 | **1.51 ± 0.06*** | **2.28 ± 0.53*** | 3.15 ± 0.04 |
| | 100 | **1.97 ± 0.14*** | **1.78 ± 0.21*** | **2.52 ± 0.14*** | ***2.04 ± 0.10**** |
| | 1000 | **0.40 ± 0.08*** | ***0.27 ± 0.04**** | ***0.36 ± 0.11**** | ***0.27 ± 0.05**** |
| Lys | 10 | 0.27 ± 0.03 | 0.77 ± 0.10 | 1.00 ± 0.82 | 2.92 ± 0.07 |
| | 100 | **1.41 ± 0.27*** | **2.75 ± 0.24*** | **2.47 ± 0.15*** | 3.11 ± 0.21 |
| | 1000 | **2.79 ± 0.15*** | **2.34 ± 0.06*** | **2.66 ± 0.37*** | 3.12 ± 0.50 |
| Met | 10 | 0.23 ± 0.02 | 0.57 ± 0.06 | ***0.18 ± 0.01**** | 2.68 ± 0.20 |

| | | | | | |
|---|---|---|---|---|---|
| | 100 | **0.31 ± 0.01*** | **1.87 ± 0.31*** | ***0.25 ± 0.10**** | ***1.84 ± 0.07**** |
| | 1000 | 0.18 ± 0.00 | ***0.27 ± 0.10**** | ***0.09 ± 0.01**** | ***0.21 ± 0.11**** |
| Phe | 10 | **0.36 ± 0.02*** | **2.19 ± 0.16*** | **2.25 ± 0.36*** | 3.32 ± 0.33 |
| | 100 | **2.69 ± 0.20*** | **2.84 ± 0.06*** | **3.37 ± 0.37*** | **3.62 ± 0.04*** |
| | 1000 | **2.88 ± 0.27*** | **2.66 ± 0.08*** | **3.83 ± 0.07*** | **4.08 ± 0.04*** |
| Pro | 10 | **0.39 ± 0.01*** | **1.00 ± 0.09*** | 0.89 ± 0.12 | 3.26 ± 0.24 |
| | 100 | **2.27 ± 0.80*** | ***0.23 ± 0.02**** | **3.53 ± 0.10*** | ***0.42 ± 0.14**** |
| | 1000 | 1.25 ± 0.65 | ***0.21 ± 0.01**** | **3.69 ± 0.12*** | ***0.58 ± 0.16**** |
| Ser | 10 | ***0.15 ± 0.01**** | **1.60 ± 0.35*** | 0.79 ± 0.03 | 3.21 ± 0.24 |
| | 100 | **2.70 ± 0.33*** | **2.96 ± 0.28*** | **3.29 ± 0.20*** | **3.91 ± 0.06*** |
| | 1000 | 0.27 ± 0.02 | ***0.45 ± 0.08**** | ***0.27 ± 0.02**** | ***0.52 ± 0.11**** |
| Thr | 10 | **0.44 ± 0.07*** | 1.28 ± 0.31 | **2.20 ± 0.24*** | 3.08 ± 0.12 |
| | 100 | **2.80 ± 0.54*** | **2.92 ± 0.01*** | **2.59 ± 0.34*** | **3.43 ± 0.08*** |
| | 1000 | 0.28 ± 0.11 | ***0.16 ± 0.02**** | 0.51 ± 0.51 | ***0.31 ± 0.15**** |
| Trp | 10 | **0.52 ± 0.06*** | **2.48 ± 0.26*** | **2.31 ± 0.12*** | 3.73 ± 0.41 |
| | 100 | **2.60 ± 0.09*** | **3.10 ± 0.14*** | **3.36 ± 0.14*** | **3.71 ± 0.12*** |
| | 1000 | **1.85 ± 0.23*** | **2.72 ± 0.09*** | **2.76 ± 0.18*** | **3.61 ± 0.27*** |
| Tyr | 10 | **0.73 ± 0.12*** | **2.67 ± 0.22*** | **2.79 ± 0.16*** | **3.76 ± 0.05*** |
| | 100 | **2.78 ± 0.33*** | **2.73 ± 0.35*** | **3.74 ± 0.12*** | 3.29 ± 0.35 |
| | 400 | **2.05 ± 0.41*** | **1.94 ± 0.03*** | **3.32 ± 0.06*** | 3.10 ± 0.11 |
| Val | 10 | 0.21 ± 0.01 | ***0.19 ± 0.00**** | ***0.20 ± 0.01**** | ***0.18 ± 0.06**** |
| | 20 | 0.23 ± 0.01 | ***0.19 ± 0.00**** | ***0.23 ± 0.01**** | ***0.19 ± 0.01**** |
| | 40 | 0.24 ± 0.01 | ***0.20 ± 0.01**** | ***0.25 ± 0.02**** | ***0.20 ± 0.02**** |

[a] Control is the condition with no addition of amino acid, and includes fifteen biological replicates from five batches, three biological replicates per batch.

* Statistically significantly different from control ($p$-value < 0.05). $P$-values are calculated using a two-tailed student's $t$-test. Significantly increased values are bolded. Significantly reduced values are bolded and italicized.

**Table S7.** Quantification of intracellular and extracellular metabolites in M9.

| | Metabolite | BW25113 | EJW3 | *P*-value |
|---|---|---|---|---|
| Intracellular | Trehalose ($\mu$g mL$^{-1}$ OD$_{600}^{-1}$) | - | - | - |
| | Glucose ($\mu$g mL$^{-1}$ OD$_{600}^{-1}$) | 770 ± 140 | 800 ± 110 | 0.706 |
| | Acetic acid ($\mu$g mL$^{-1}$ OD$_{600}^{-1}$) | 650 ± 90 | ***390 ± 80*** | 0.000* |
| | Asp ($\mu$g mL$^{-1}$ OD$_{600}^{-1}$) | - | - | - |
| | Glu ($\mu$g mL$^{-1}$ OD$_{600}^{-1}$) | 187.72 ± 35.94 | 212.37 ± 27.2 | 0.210 |
| | Asn Ser ($\mu$g mL$^{-1}$ OD$_{600}^{-1}$) | 9.45 ± 0.75 | 10.11 ± 0.94 | 0.204 |
| | Gln Gly His Thr ($\mu$g mL$^{-1}$ OD$_{600}^{-1}$) | 10.82 ± 0.67 | 10.14 ± 2.49 | 0.534 |
| | Ala ($\mu$g mL$^{-1}$ OD$_{600}^{-1}$) | 81.18 ± 16.55 | 83.71 ± 3.15 | 0.721 |
| | Arg ($\mu$g mL$^{-1}$ OD$_{600}^{-1}$) | - | 4.78 ± 7.41 | 0.145 |
| | Tyr ($\mu$g mL$^{-1}$ OD$_{600}^{-1}$) | - | 4.10 ± 10.04 | 0.341 |
| | Val ($\mu$g mL$^{-1}$ OD$_{600}^{-1}$) | - | - | - |
| | Met ($\mu$g mL$^{-1}$ OD$_{600}^{-1}$) | 55.11 ± 6.01 | **74.52 ± 10.88** | 0.003* |
| | Trp ($\mu$g mL$^{-1}$ OD$_{600}^{-1}$) | - | - | - |
| | Phe Ile ($\mu$g mL$^{-1}$ OD$_{600}^{-1}$) | - | - | - |
| | Leu ($\mu$g mL$^{-1}$ OD$_{600}^{-1}$) | 3.83 ± 9.38 | 17.56 ± 13.81 | 0.072 |
| | Lys ($\mu$g mL$^{-1}$ OD$_{600}^{-1}$) | 6.95 ± 10.78 | 7.08 ± 11.24 | 0.983 |
| | Pro ($\mu$g mL$^{-1}$ OD$_{600}^{-1}$) | 61.64 ± 6.12 | **75.06 ± 1.95** | 0.000* |
| Extracellular | Trehalose ($\mu$g mL$^{-1}$ OD$_{600}^{-1}$) | - | - | - |
| | Glucose ($\mu$g mL$^{-1}$ OD$_{600}^{-1}$) | 5280 ± 300 | 5380 ± 140 | 0.466 |
| | Acetic acid ($\mu$g mL$^{-1}$ OD$_{600}^{-1}$) | 280 ± 20 | **370 ± 10** | 0.000* |
| | Asp ($\mu$g mL$^{-1}$OD$_{600}^{-1}$) | - | - | - |
| | Glu ($\mu$g mL$^{-1}$OD$_{600}^{-1}$) | - | 6.42 ± 12.41 | 0.234 |
| | Asn Ser ($\mu$g mL$^{-1}$ OD$_{600}^{-1}$) | - | - | - |
| | Gln Gly His Thr ($\mu$g mL$^{-1}$ OD$_{600}^{-1}$) | - | - | - |
| | Ala ($\mu$g mL$^{-1}$ OD$_{600}^{-1}$) | 10.86 ± 6.33 | ***1.93 ± 4.72*** | 0.020* |
| | Arg ($\mu$g mL$^{-1}$ OD$_{600}^{-1}$) | 2.95 ± 7.21 | - | 0.341 |
| | Tyr ($\mu$g mL$^{-1}$ OD$_{600}^{-1}$) | 26.01 ± 14.8 | **45.14 ± 5.72** | 0.014* |
| | Val ($\mu$g mL$^{-1}$ OD$_{600}^{-1}$) | - | - | - |
| | Met ($\mu$g mL$^{-1}$ OD$_{600}^{-1}$) | 3.92 ± 9.60 | **41.35 ± 11.43** | 0.000* |
| | Trp ($\mu$g mL$^{-1}$ OD$_{600}^{-1}$) | 24.85 ± 60.86 | **131.60 ± 90.56** | 0.038* |
| | Phe Ile ($\mu$g mL$^{-1}$ OD$_{600}^{-1}$) | 89.07 ± 45.53 | ***20.37 ± 49.90*** | 0.032* |
| | Leu ($\mu$g mL$^{-1}$ OD$_{600}^{-1}$) | - | - | - |
| | Lys ($\mu$g mL$^{-1}$ OD$_{600}^{-1}$) | - | - | - |
| | Pro ($\mu$g mL$^{-1}$ OD$_{600}^{-1}$) | 516.13 ± 35.09 | **776.02 ± 117.02** | 0.000* |

- Not detectible. * Statistically significantly different between BW25113 and EJW3 ($p$-values < 0.05). Values bolded are significantly higher in EJW3. Values bolded and italicized are significantly lower in EJW3. $P$-values are calculated by using a two-tailed student's $t$-test with six biological replicates.

**Table S8.** Quantification of intracellular and extracellular metabolites in M9 supplemented with 0.6 M NaCl.

| | Metabolite | BW25113 | EJW3 | *P*-value |
|---|---|---|---|---|
| Intracellular | Trehalose ($\mu g\ mL^{-1}\ OD_{600}^{-1}$) | 1190 ± 190 | **1630 ± 220** | 0.005* |
| | Glucose ($\mu g\ mL^{-1}\ OD_{600}^{-1}$) | 1050 ± 80 | 1030 ± 180 | 0.797 |
| | Acetic acid ($\mu g\ mL^{-1}\ OD_{600}^{-1}$) | 270 ± 140 | 370 ± 50 | 0.137 |
| | Asp ($\mu g\ mL^{-1}\ OD_{600}^{-1}$) | - | - | - |
| | Glu ($\mu g\ mL^{-1}\ OD_{600}^{-1}$) | 565.50 ± 44.39 | **698.75 ± 82.47** | 0.006* |
| | Asn Ser ($\mu g\ mL^{-1}\ OD_{600}^{-1}$) | 1.02 ± 2.50 | **6.74 ± 3.71** | 0.011* |
| | Gln Gly His Thr ($\mu g\ mL^{-1}\ OD_{600}^{-1}$) | 8.64 ± 1.59 | 9.69 ± 4.83 | 0.623 |
| | Ala ($\mu g\ mL^{-1}\ OD_{600}^{-1}$) | 38.49 ± 9.03 | **71.58 ± 13.51** | 0.001* |
| | Arg ($\mu g\ mL^{-1}\ OD_{600}^{-1}$) | 28.79 ± 2.73 | **44.87 ± 6.42** | 0.000* |
| | Tyr ($\mu g\ mL^{-1}\ OD_{600}^{-1}$) | - | - | - |
| | Val ($\mu g\ mL^{-1}\ OD_{600}^{-1}$) | - | 0.44 ± 1.08 | 0.341 |
| | Met ($\mu g\ mL^{-1}\ OD_{600}^{-1}$) | 21.29 ± 6.35 | **30.55 ± 2.53** | 0.008* |
| | Trp ($\mu g\ mL^{-1}\ OD_{600}^{-1}$) | - | - | - |
| | Phe Ile ($\mu g\ mL^{-1}\ OD_{600}^{-1}$) | - | 3.24 ± 5.27 | 0.164 |
| | Leu ($\mu g\ mL^{-1}\ OD_{600}^{-1}$) | - | - | - |
| | Lys ($\mu g\ mL^{-1}\ OD_{600}^{-1}$) | - | 9.08 ± 10.05 | 0.051 |
| | Pro ($\mu g\ mL^{-1}\ OD_{600}^{-1}$) | 94.31 ± 6.14 | 107.31 ± 18.37 | 0.131 |
| Extracellular | Trehalose ($\mu g\ mL^{-1}\ OD_{600}^{-1}$) | - | - | - |
| | Glucose ($\mu g\ mL^{-1}\ OD_{600}^{-1}$) | 4950 ± 770 | 4600 ± 1000 | 0.518 |
| | Acetic acid ($\mu g\ mL^{-1}\ OD_{600}^{-1}$) | 260 ± 20 | **360 ± 20** | 0.000* |
| | Asp ($\mu g\ mL^{-1}\ OD_{600}^{-1}$) | - | - | - |
| | Glu ($\mu g\ mL^{-1}\ OD_{600}^{-1}$) | 234.82 ± 15.44 | ***88.05 ± 19.38*** | 0.000* |
| | Asn Ser ($\mu g\ mL^{-1}\ OD_{600}^{-1}$) | - | - | - |
| | Gln Gly His Thr ($\mu g\ mL^{-1}\ OD_{600}^{-1}$) | - | - | - |
| | Ala ($\mu g\ mL^{-1}\ OD_{600}^{-1}$) | - | - | - |
| | Arg ($\mu g\ mL^{-1}\ OD_{600}^{-1}$) | - | 7.90 ± 12.34 | 0.148 |
| | Tyr ($\mu g\ mL^{-1}\ OD_{600}^{-1}$) | 41.73 ± 6.31 | ***16.05 ± 24.95*** | 0.035* |
| | Val ($\mu g\ mL^{-1}\ OD_{600}^{-1}$) | - | - | - |
| | Met ($\mu g\ mL^{-1}\ OD_{600}^{-1}$) | 40.03 ± 8.39 | 38.80 ± 18.26 | 0.884 |
| | Trp ($\mu g\ mL^{-1}\ OD_{600}^{-1}$) | 88.49 ± 30.23 | ***20.22 ± 49.52*** | 0.016* |
| | Phe Ile ($\mu g\ mL^{-1}\ OD_{600}^{-1}$) | 0.86 ± 2.10 | **85.07 ± 46.36** | 0.001* |
| | Leu ($\mu g\ mL^{-1}\ OD_{600}^{-1}$) | - | - | - |
| | Lys ($\mu g\ mL^{-1}\ OD_{600}^{-1}$) | - | - | - |
| | Pro ($\mu g\ mL^{-1}\ OD_{600}^{-1}$) | 747.75 ± 120.88 | 728.90 ± 156.43 | 0.820 |

- Not detectible. * Statistically significantly different between BW25113 and EJW3 ($p$-values < 0.05). Values bolded are significantly higher in EJW3. Values bolded and italicized are significantly lower in EJW3. $P$-values are calculated by using a two-tailed student's $t$-test with six biological replicates.

**Table S9.** Upregulated genes selected for validation for their roles in osmotic tolerance.

| ID | Function |
|---|---|
| b0260 | CP4-6 prophage; putative S-methylmethionine transporter (*mmuP*) |
| b3939 | cystathionine gamma-synthase, PLP-dependent (*metB*) |
| b4013 | homoserine O-transsuccinylase (*metA*) |
| b3828 | methionine biosynthesis regulon transcriptional regulator (*metR*) |
| b2942 | S-adenosylmethionine synthetase (*metK*) |
| b2421 | cysteine synthase B (O-acetylserine sulfhydrolase B) (*cysM*) |
| b2366 | D-serine dehydratase (*dsdA*) |
| b4131 | lysine decarboxylase, acid-inducible (*cadA*) |
| b0402 | proline-specific permease (*proY*) |
| b1386 | tyramine oxidase, copper-requiring (*tynA*) |

**Table S10**. Gene ontology analysis in M9 supplemented with 0.6 M NaCl.

| | **Category** | **Term** | **Count** | **%** | ***p*-value** |
|---|---|---|---|---|---|
| Upregulated (BW25113) | GOTERM_BP_DIRECT | GO:0009086~methionine biosynthetic process | 4 | 5.405 | 0.001 |
| | UP_KEYWORDS | Methionine biosynthesis | 4 | 5.405 | 0.002 |
| | UP_KEYWORDS | Pyridoxal phosphate | 6 | 8.108 | 0.004 |
| Downregulated (BW25113) | UP_KEYWORDS | Transmembrane helix | 21 | 41.176 | 0.001 |
| | UP_KEYWORDS | Cell membrane | 23 | 45.098 | 0.001 |
| | UP_SEQ_FEATURE | transmembrane region | 21 | 41.176 | 0.001 |
| | UP_KEYWORDS | Transmembrane | 21 | 41.176 | 0.001 |
| | UP_SEQ_FEATURE | topological domain:Cytoplasmic | 16 | 31.373 | 0.001 |
| | UP_SEQ_FEATURE | topological domain:Periplasmic | 16 | 31.373 | 0.002 |
| | UP_KEYWORDS | Membrane | 23 | 45.098 | 0.004 |
| | UP_KEYWORDS | Cell inner membrane | 19 | 37.255 | 0.005 |
| | COG_ONTOLOGY | Inorganic ion transport and metabolism | 4 | 7.843 | 0.006 |
| | GOTERM_BP_DIRECT | GO:0006814~sodium ion transport | 3 | 5.882 | 0.009 |
| | GOTERM_CC_DIRECT | GO:0005886~plasma membrane | 21 | 41.176 | 0.012 |
| Upregulated (MG1655) | KEGG_PATHWAY | eco00780:Biotin metabolism | 4 | 3.636 | 0.001 |
| Downregulated (MG1655) | KEGG_PATHWAY | eco00190:Oxidative phosphorylation | 19 | 13.571 | 0.000 |
| | UP_KEYWORDS | Quinone | 10 | 7.143 | 0.000 |
| | UP_KEYWORDS | Ubiquinone | 10 | 7.143 | 0.000 |
| | GOTERM_CC_DIRECT | GO:0045272~plasma membrane respiratory chain complex I | 10 | 7.143 | 0.000 |
| | GOTERM_CC_DIRECT | GO:0030964~NADH dehydrogenase complex | 10 | 7.143 | 0.000 |
| | GOTERM_BP_DIRECT | GO:0009060~aerobic respiration | 11 | 7.857 | 0.000 |
| | GOTERM_MF_DIRECT | GO:0048038~quinone binding | 9 | 6.429 | 0.000 |

| | | | | |
|---|---|---|---|---|
| GOTERM_MF_DIRECT | GO:0003954~NADH dehydrogenase activity | 10 | 7.143 | 0.000 |
| UP_KEYWORDS | Ligase | 15 | 10.714 | 0.000 |
| GOTERM_MF_DIRECT | GO:0008137~NADH dehydrogenase (ubiquinone) activity | 8 | 5.714 | 0.000 |
| UP_KEYWORDS | Oxidoreductase | 28 | 20 | 0.000 |
| UP_KEYWORDS | Enterobactin biosynthesis | 5 | 3.571 | 0.000 |
| GOTERM_BP_DIRECT | GO:0006099~tricarboxylic acid cycle | 8 | 5.714 | 0.000 |
| UP_KEYWORDS | Tricarboxylic acid cycle | 7 | 5 | 0.000 |
| KEGG_PATHWAY | eco00020:Citrate cycle (TCA cycle) | 9 | 6.429 | 0.000 |
| UP_KEYWORDS | Transport | 43 | 30.714 | 0.000 |
| GOTERM_BP_DIRECT | GO:0009239~enterobactin biosynthetic process | 5 | 3.571 | 0.000 |
| GOTERM_BP_DIRECT | GO:0042773~ATP synthesis coupled electron transport | 5 | 3.571 | 0.000 |
| GOTERM_BP_DIRECT | GO:0015990~electron transport coupled proton transport | 5 | 3.571 | 0.000 |
| KEGG_PATHWAY | eco02040:Flagellar assembly | 10 | 7.143 | 0.000 |
| KEGG_PATHWAY | eco01053:Biosynthesis of siderophore group nonribosomal peptides | 5 | 3.571 | 0.000 |
| UP_KEYWORDS | Cell inner membrane | 49 | 35 | 0.000 |
| KEGG_PATHWAY | eco00250:Alanine, aspartate and glutamate metabolism | 9 | 6.429 | 0.000 |
| UP_KEYWORDS | Pyrimidine biosynthesis | 5 | 3.571 | 0.000 |
| GOTERM_MF_DIRECT | GO:0030976~thiamine pyrophosphate binding | 5 | 3.571 | 0.000 |
| UP_KEYWORDS | Cell membrane | 54 | 38.571 | 0.000 |
| UP_KEYWORDS | Membrane | 58 | 41.429 | 0.000 |
| UP_KEYWORDS | NAD | 14 | 10 | 0.001 |
| GOTERM_MF_DIRECT | GO:0015421~oligopeptide-transporting ATPase activity | 4 | 2.857 | 0.001 |
| GOTERM_BP_DIRECT | GO:0044205~'de novo' UMP biosynthetic process | 4 | 2.857 | 0.002 |

| | | | | |
|---|---|---|---|---|
| UP_KEYWORDS | Bacterial flagellum biogenesis | 5 | 3.571 | 0.002 |
| UP_KEYWORDS | Purine biosynthesis | 5 | 3.571 | 0.002 |
| UP_KEYWORDS | Nucleotide-binding | 29 | 20.714 | 0.002 |
| KEGG_PATHWAY | eco01100:Metabolic pathways | 55 | 39.286 | 0.003 |
| GOTERM_BP_DIRECT | GO:0019646~aerobic electron transport chain | 4 | 2.857 | 0.004 |
| UP_KEYWORDS | Bacterial flagellum | 5 | 3.571 | 0.004 |
| GOTERM_MF_DIRECT | GO:0042936~dipeptide transporter activity | 4 | 2.857 | 0.004 |
| UP_KEYWORDS | ATP-binding | 25 | 17.857 | 0.004 |
| GOTERM_MF_DIRECT | GO:0047527~2,3-dihydroxybenzoate-serine ligase activity | 3 | 2.143 | 0.004 |
| GOTERM_BP_DIRECT | GO:0044781~bacterial-type flagellum organization | 4 | 2.857 | 0.005 |
| GOTERM_BP_DIRECT | GO:0042938~dipeptide transport | 4 | 2.857 | 0.005 |
| UP_KEYWORDS | Thiamine pyrophosphate | 4 | 2.857 | 0.006 |
| UP_KEYWORDS | Glutamine amidotransferase | 4 | 2.857 | 0.006 |
| GOTERM_CC_DIRECT | GO:0009424~bacterial-type flagellum hook | 4 | 2.857 | 0.006 |
| UP_SEQ_FEATURE | topological domain:Cytoplasmic | 31 | 22.143 | 0.007 |
| UP_SEQ_FEATURE | topological domain:Periplasmic | 31 | 22.143 | 0.007 |
| GOTERM_BP_DIRECT | GO:0015796~galactitol transport | 3 | 2.143 | 0.008 |
| GOTERM_MF_DIRECT | GO:0090584~protein-phosphocysteine-galactitol-phosphotransferase system transporter activity | 3 | 2.143 | 0.008 |

64

## 3. Supplementary Figures

BW25113 M9 0h

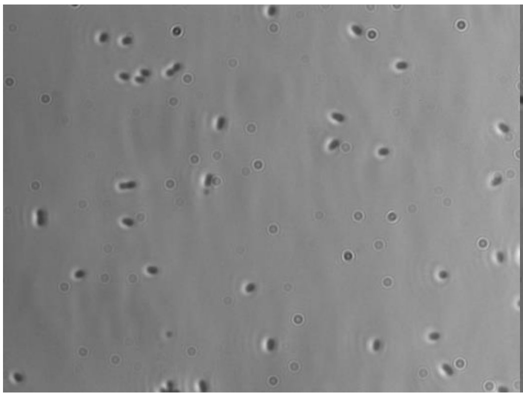

EJW3 M9 0h

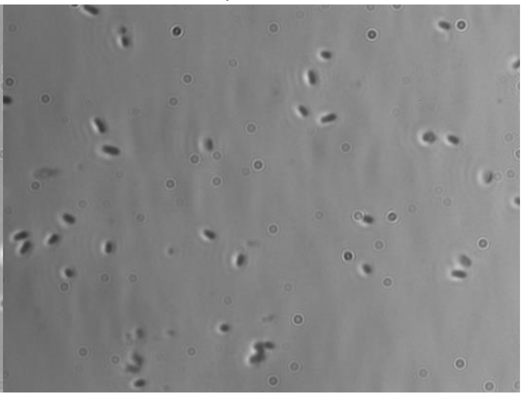

BW25113 M9 3h

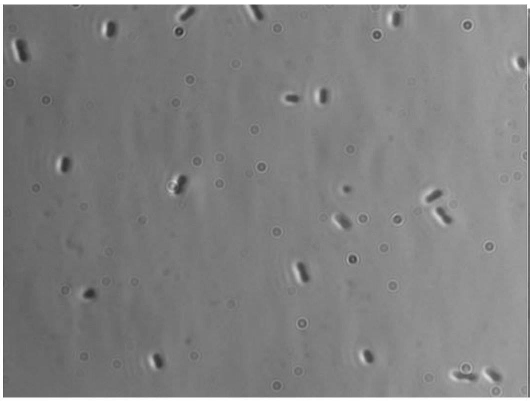

EJW3 M9 3h

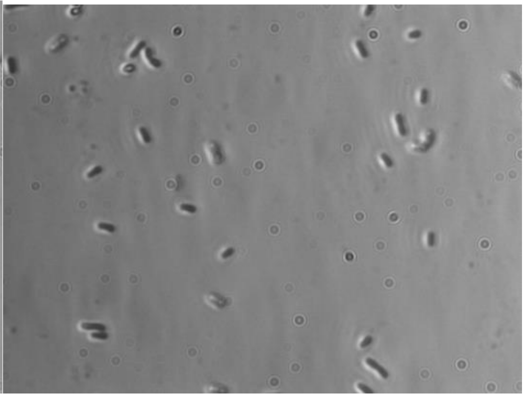

BW25113 M9 6h

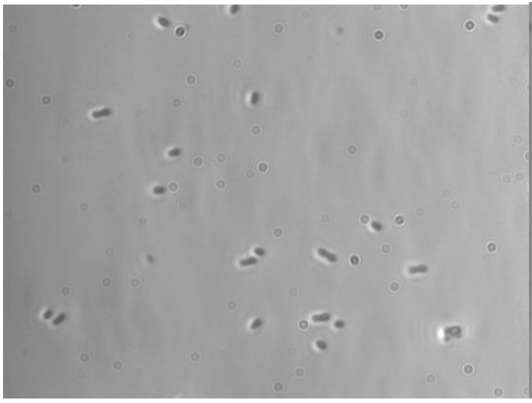

EJW3 M9 6h

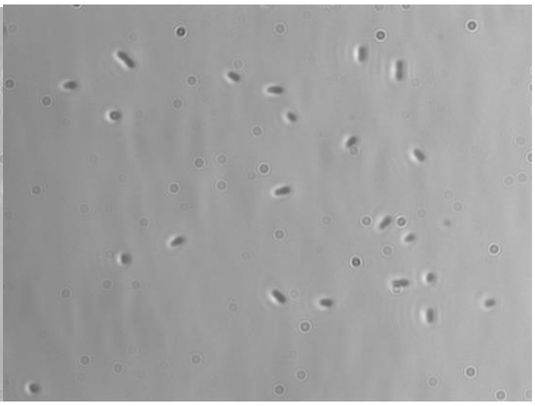

BW25113 0.6 M NaCl 3h

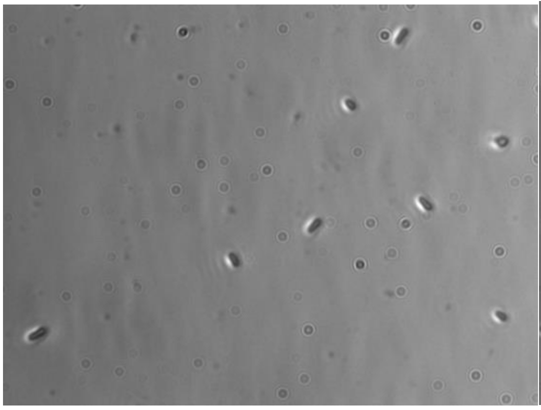

EJW3 0.6 M NaCl 3h

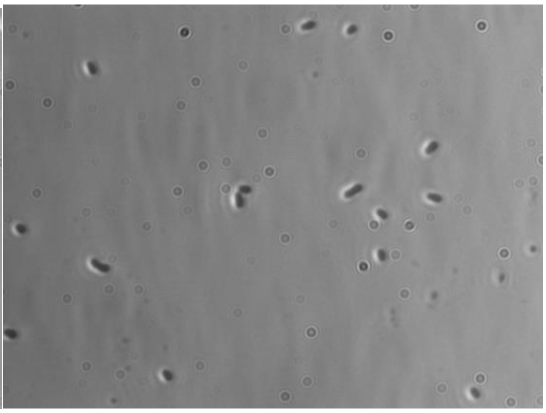

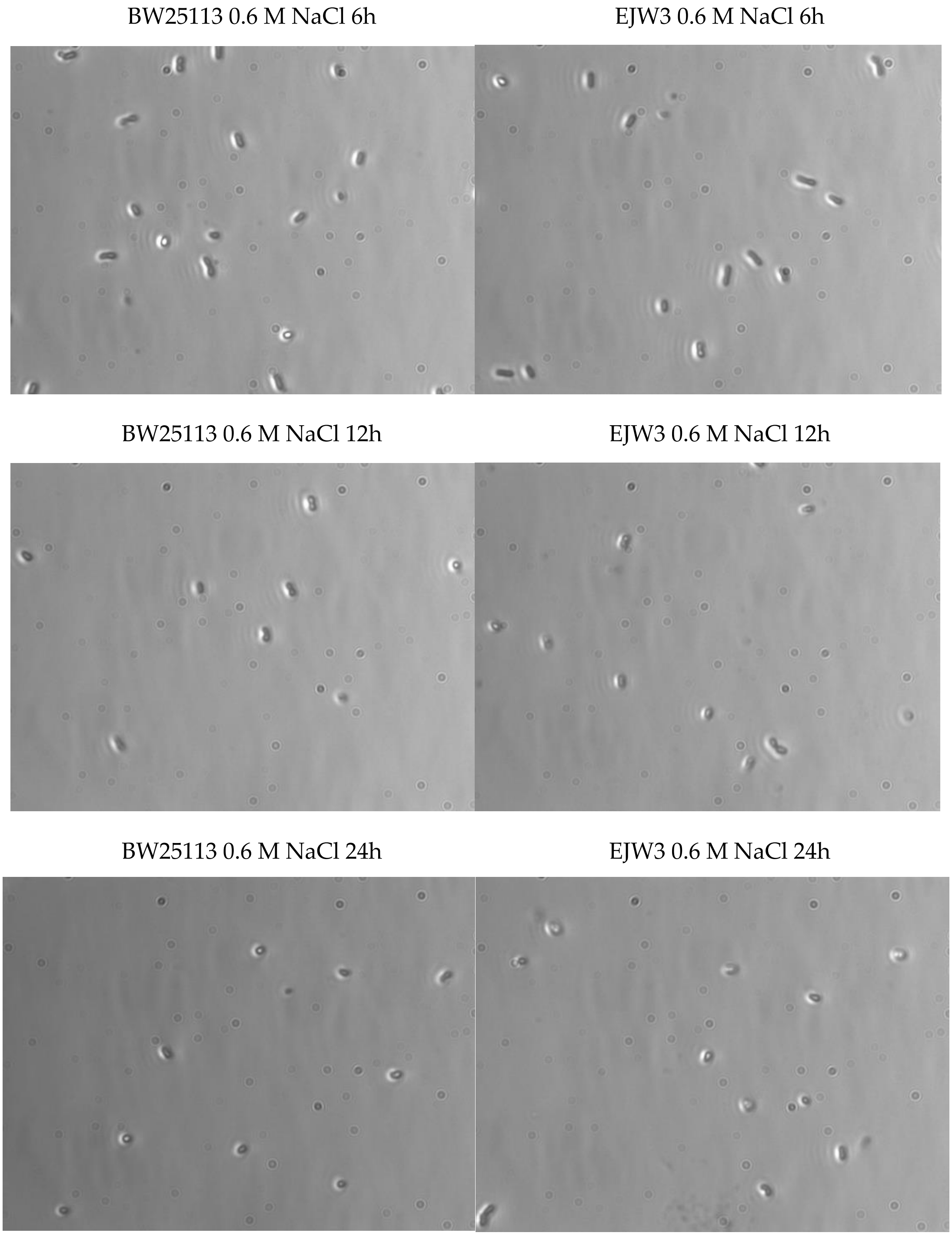


**Figure. S1.** Light microscopy of cells in the presence or absence of hyperosmotic stress.

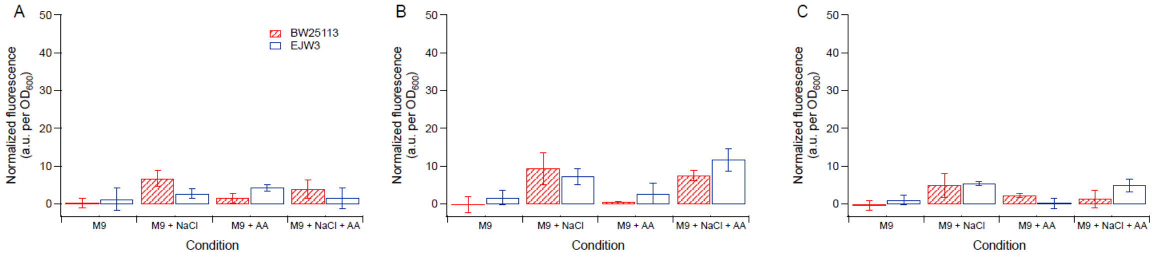


**Figure. S2.** Membrane integrity assay using PI staining. (A) lag phase cells. (B) Early stationary phase cells. (C) Late stationary phase cells. NaCl: 0.7 M NaCl. AA: 10 mM acetic acid. Error bars are standard deviations.

## References

1. Rabinowitz, J.D.; Kimball, E. Acidic acetonitrile for cellular metabolome extraction from *Escherichia coli*. Anal Chem 2007, 79, 6167-6173.
2. Henderson, J.W.; Brooks, A. Improved amino acid methods using Agilent ZORBAX Eclipse Plus C18 columns for a variety of Agilent LC instrumentation and separation goals. Agilent Technologies 2010.
